# Genome‐wide DNA methylation analysis identifies potent CpG signature for temzolomide response in non‐G‐CIMP glioblastomas with unmethylated *MGMT* promoter: *MGMT* ‐dependent roles of *GPR81*

**DOI:** 10.1111/cns.14465

**Published:** 2023-10-13

**Authors:** Bao‐Bao Liang, Yu‐Hong Wang, Jing‐Jing Huang, Shuai Lin, Guo‐Chao Mao, Zhang‐Jian Zhou, Wan‐Jun Yan, Chang‐You Shan, Hui‐Zi Wu, Amandine Etcheverry, Ya‐Long He, Fang‐Fang Liu, Hua‐Feng Kang, An‐An Yin, Shu‐Qun Zhang

**Affiliations:** ^1^ Department of Oncology The Second Affiliated Hospital of Xi'an Jiaotong University Xi'an Shaanxi China; ^2^ The Emergency Department The Seventh Medical Center of Chinese PLA General Hospital Beijing China; ^3^ Department of Pediatric Surgery The Second Affiliated Hospital of Xi'an Jiaotong University Xi'an Shaanxi China; ^4^ CNRS, UMR 6290, Institut de Génétique et Développement de Rennes (IGdR) Rennes France; ^5^ Department of Neurosurgery, Xijing Hospital Air Force Medical University Xi'an China; ^6^ Institute of Neurosciences, College of Basic Medicine Air Force Medical University Xi'an China; ^7^ Department of Biochemistry and Molecular Biology Air Force Medical University Xi'an China; ^8^ Department of Plastic and Reconstructive Surgery, Xijing Hospital Air Force Medical University Xi'an China

**Keywords:** DNA methylation, glioblastoma, predictive biomarker, temozolomide, unmethylated *MGMT*

## Abstract

**Purposes:**

To identify potent DNA methylation candidates that could predict response to temozolomide (TMZ) in glioblastomas (GBMs) that do not have glioma‐CpGs island methylator phenotype (G‐CIMP) but have an unmethylated promoter of O‐6‐methylguanine‐DNA methyltransferase (un*MGMT*).

**Methods:**

The discovery‐validation approach was planned incorporating a series of G‐CIMP−/un*MGMT* GBM cohorts with DNA methylation microarray data and clinical information, to construct multi‐CpG prediction models. Different bioinformatic and experimental analyses were performed for biological exploration.

**Results:**

By analyzing discovery sets with radiotherapy (RT) plus TMZ versus RT alone, we identified a panel of 64 TMZ efficacy‐related CpGs, from which a 10‐CpG risk signature was further constructed. Both the 64‐CpG panel and the 10‐CpG risk signature were validated showing significant correlations with overall survival of G‐CIMP−/un*MGMT* GBMs when treated with RT/TMZ, rather than RT alone. The 10‐CpG risk signature was further observed for aiding TMZ choice by distinguishing differential outcomes to RT/TMZ versus RT within each risk subgroup. Functional studies on *GPR81*, the gene harboring one of the 10 CpGs, indicated its distinct impacts on TMZ resistance in GBM cells, which may be dependent on the status of *MGMT* expression.

**Conclusions:**

The 64 TMZ efficacy‐related CpGs and in particular the 10‐CpG risk signature may serve as promising predictive biomarker candidates for guiding optimal usage of TMZ in G‐CIMP−/un*MGMT* GBMs.

## Graphical abstract

By analyzing DNA methylation microarray data and clinical information of glioblastomas (GBMs) that do not have glioma‐CpGs island methylator phenotype (G‐CIMP) but have an unmethylated promoter of O‐6‐methylguanine‐DNA methyltransferase (unMGMT), a panel of 64 CpGs and a 10‐CpG risk score signature were discovered and validated with specific linkage to temozolomide (TMZ) efficacy. Experimental data provided biological clues behind the risk signature. The 64 CpGs and in particular the 10‐CpG signature may serve as promising predictive biomarker candidates for guiding optimal usage of TMZ in GBMs with G‐CIMP‐ and un*MGMT*.

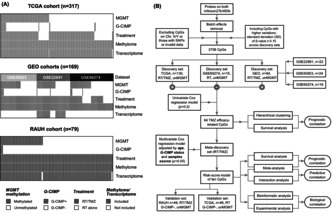

## 1 INTRODUCTION

Glioblastomas (GBMs) are the most frequent and devastating brain malignancy with a reported median survival of only 12 ~ 15 months.^1^ Temozolomide (TMZ) stands for the most effective adjuvant chemotherapy for GBMs.^2^ It can alkylate genomic DNA of tumor cells at multiple sites and induce cell cycle arrest and apoptosis.^2^ Unfortunately, the deadly GBMs, characteristic of high inter‐tumoral molecular heterogeneity, variably responded to TMZ.^2^ A considerable number of patients did not benefit from the treatment of TMZ, and some even suffer from its high costs and adverse effects.^3^, ^4^, ^5^

The promoter methylation status of the O‐6‐methylguanine‐DNA methyltransferase (*MGMT*) gene, encoding a DNA repair enzyme that conferred major resistance to alkylating agents, is by far the most informative predictive biomarker for TMZ response.^3^, ^4^, ^5^ TMZ was generally beneficial to GBMs that harbor a methylated (me)*MGMT* promoter and consequently have a low expression of MGMT. However, TMZ was not that sensitive to GBMs harboring an unmethylated (un)*MGMT* promoter and generally a high expression of MGMT and tended to yield variable outcomes in those tumors. *MGMT* testing may thus have limited use in guiding TMZ choice for GBM patients and especially those with un*MGMT* GBMs.^6^, ^7^, ^8^ Therefore, discovering powerful biomarkers that are predictive of TMZ response for subpopulation with TMZ‐resistant un*MGMT* tumors can be clinically useful.^9^, ^10^

In 2010, The Cancer Genome Atlas (TCGA) research group reported a novel and distinct subtype of GBMs with glioma GpGs island methylator phenotype (G‐CIMP)^11^; the subtype was characterized by exclusive mutations in isocitrate dehydrogenase (IDH) gene, concordant DNA hypermethylation at CpG islands throughout genome, good sensitivity to radio‐chemotherapy, and considerably favorable prognosis.^11^, ^12^, ^13^, ^14^, ^15^, ^16^, ^17^, ^18^ Because of the small proportion (10% of all GBMs) and the very distinct molecular and clinical features, the G‐CIMP subtype was excluded, and we mainly focused on the majority of GBMs that do not have G‐CIMP. In this study, by integrating genome‐wide DNA methylation microarray data and clinical information, we identified a panel of 64 CpG candidates with potential linkage to TMZ efficacy in G‐CIMP‐ GBMs with un*MGMT* promoter, from which a 10‐CpG risk signature was constructed and validated to robustly and stably predict TMZ response. Bioinformatic and in vitro experimental analyses further provided biological insights into the 10‐CpG predictive signature.

## 2 METHODS

### 2.1 Determination of G‐CIMP and *MGMT* promoter methylation status

The G‐CIMP status and promoter methylation status of *MGMT* were defined using DNA methylation data from Illumina HumanMethylation27k/450 k microarrays (Illumina Inc.), as previously reported.^19^, ^20^ Within the Illumina platform, methylation signal of each CpG was summarized as *β* value which provides a continuous and quantitative measurement of DNA methylation ranging from 0 (completely unmethylated) to 1 (completely methylated).^21^ The *β* value was commonly used for intuitive biological interpretation.^21^ When statistical analysis was employed, *β* value was transformed to M value, which has a Logit transformation relationship with *β* value.^21^ The G‐CIMP status was determined by *K*‐means (*k* = 3) clustering using the 1,503 featured probes as previously described.^19^ The *MGMT* promoter methylation was determined by a logistical regression model using two Illumina probes (cg12434587 and cg12981137).^20^ The logistical regression formula was calculated as logit (*y*) = 4.3215 + 0.5271 *×* M‐value of cg12434587 + 0.9265 × M‐value of cg12981137, with a cutoff of 0.358 for stratifying methylated and unmethylated tumors.^20^

### 2.2 GBM datasets of clinical samples and cell lines

**FIGURE 1:**
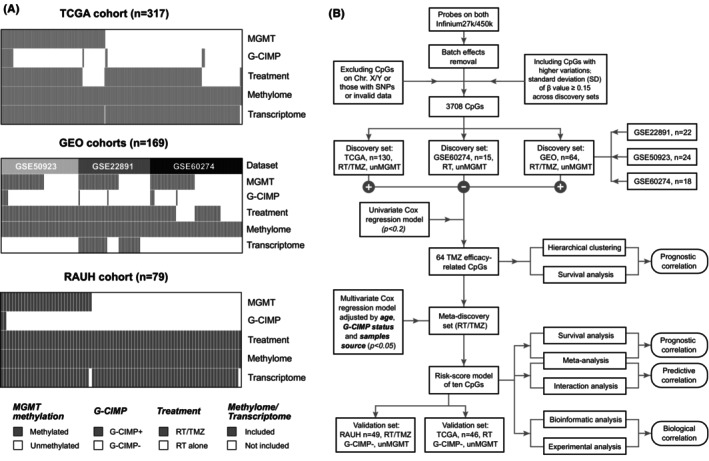
Patient dataset information and study workflow; (A) molecular subtype, available molecular/clinical data, and sample size of included patient datasets; (B) schematic diagram for searching and validating TMZ efficacy‐related CpG candidates and a 10‐CpG signature.

Molecular microarray data and clinical information of primary GBMs were downloaded from public databases, including one cohort from TCGA (*n* = 317)^22^ and three cohorts from Gene expression omnibus (GEO; GSE22891,^23^ *n* = 50; GSE50923, *n* = 54^24^; and GSE60274, *n* = 64^25^). A French dataset of 79 primary GBMs from Rennes and Angers University Hospitals (RAUH) were provided by Prof. Amandine Etcheverry.^26^ In addition, non‐tumor brains (NTBs) from TCGA^22^ and GSE63347^27^ were included for comparative analysis. All samples had DNA methylation data profiled by Infinium HumanMethylation 27 k or 450 k chips, and some had paired gene expression data profiled by a variety of transcriptome microarrays.^22^, ^23^, ^24^, ^25^, ^26^ The availability of molecular and clinical data for each sample was presented in Figure 1A. Finally, DNA methylation (Infinium HumanMethylation 450 k) and gene expression (Affymetrix HumanGenome U219‐96) microarray data of 36 GBM cell lines were downloaded from GSE68379.^28^ More information on microarray platform and data processing for each dataset can be referred to the original reports.^22^, ^23^, ^24^, ^25^, ^26^, ^27^, ^28^

### 2.3 CpG selection and model construction

The discovery‐validation approach was utilized to identify CpG candidates with potential linkage to TMZ efficacy. The workflow for study design is shown in Figure 1B. In this study, RT/TMZ‐treated un*MGMT* GBMs from TCGA and GEO (GSE22891, GSE50923, and GSE60274) and RT‐treated un*MGMT* tumors from GSE60274 were preset as discovery sets. RT/TMZ‐treated un*MGMT* GBMs from RAUH and RT‐treated un*MGMT* tumors from TCGA were preset as validation sets (Figure 1B).

Initial CpG selection was done as reported in our previous study.^29^ Batch effects across datasets were adjusted using M‐value transformation^21^ and the Empirical Bayes method (ComBat module, GenePattern).^30^ CpGs with higher variability in methylation levels (standard deviation [SD] of *β* value in discovery sets ≥0.15) were kept, and their M‐values were used to correlate with overall survival (OS) in each discovery set treated with either RT/TMZ or RT alone. After removing inconsistent results from univariate Cox regression analyses, a panel of 64 CpGs was identified as TMZ efficacy‐related candidates. To further reduce data dimensionality, the 64‐CpG methylation data were incorporated into a multivariate Cox regression model using Likelihood Ratio (LR) and Forward selection approach, where age, sample source, and G‐CIMP status were together adjusted. Finally, we identified a total of ten CpGs to construct a RISK‐score formula, which is the sum of the M‐value of each CpG weighted by its corresponding multivariate Cox coefficient. The median RISK score value in the RT/TMZ‐treated discovery sets was pre‐defined as the cutoff for low‐risk and high‐risk groups.

### 2.4 Gene set enrichment analysis

Gene set enrichment analysis (GESA) was run through the Gene Ontology Gene Set collection from Molecular Signatures Database (MSigDB), to evaluate the biological profiles of the risk subgroups, with both nominal *p*‐value ≤0.05 and false discovery rate (FDR) ≤ 0.25 as statistical significance.^31^ The proportion of 28 tumor‐infiltrating immune cell (TIIC) types in tumor bulks was estimated using single‐sample GSEA (ssGSEA) approach and the 782‐gene signature reported by Charoentong et al.^32^ The abundance of each TIIC type was summarized as normalized enrichment scores (NES).

### 2.5 Immunohistochemistry (IHC) staining

Formalin‐fixed paraffin‐embedded (FFPE) samples of 10 primary GBMs and 3 NTBs from patients with traumatic brain injury were collected from the Department of Neurosurgery, Xijing Hospital. FFPE samples were employed for IHC staining with anti‐GPR81 antibody (Abcam, #ab106942) or anti‐MGMT antibody (Proteintech, #17195‐1‐AP). The intensity and percentage of positive cells were evaluated in at least five separate fields at × 400 magnification. Immunoreactivity was scored as follows: 0, no staining; 1, weak staining in <50% cells; 2, weak staining in ≥h50% cells; 3, strong staining in <50% cells; and 4, strong staining in ≥50% cells. The scores were evaluated by two independent researchers, and disputes were resolved through discussion. All patients provided written informed consent, and this study was approved by the Institutional Review Board.

### 2.6 Cell culture and treatment

Two GBM cell lines (A172 and T98G) were obtained from American Type Culture Collection and grown in Dulbecco's modified Eagle's medium containing 10% fetal bovine serum at 37°C in 5% CO_2_. The cells were treated with TMZ (MedChemExpress, #HY‐17364) at indicated concentrations for 48 h. TMZ was dissolved in dimethyl sulfoxide (DMSO, Sigma‐Aldrich).

### 2.7 Plasmids and cell transfection

For in vitro gene silencing, RNA interference using plasmids expressing human short hairpin RNAs (shRNAs) was performed. Plasmids containing shRNAs targeting *GRP81* (sh*GPR81*) or *MGMT* (sh*MGMT*), and scramble shRNAs (shControl) were purchased from GeneChem. For in vitro gene overexpression, plasmids containing the *MGMT* gene were also obtained (GeneChem). Cell transfection was done using Lipofectamine® 2000 reagent (Invitrogen). Transfection efficiency was verified by quantitative real‐time PCR (qRT‐PCR).

### 2.8 TMZ cytotoxicity assay

After plasmid transfection, T98G and A172 cells were seeded on 96 well plates (5000 cells per well) and were treated with TMZ at the final concentrations of 7.5, 15, 30, 60, 120, 240, and 480 μM for 48 h. CCK‐8 reagent was added to wells (10 μL/well) and incubated at 37°C for 1 h. The absorbance at 450 nm was measured for calculating the half maximal inhibitory concentration (IC_50_).

### 2.9 qRT‐PCR

The total RNA was extracted using TRIzol reagent (Shanghai Pufei Biotech) and reverse‐transcribed with M‐MLV RT kit (Promega) according to the manufacturer's instructions. PCR amplification was performed with SYBR Master Mixture (Takara) using LighterCycler 480 II System (Rcoche). The mRNA ratio of a target gene to GAPDH was calculated using the 2^−ΔΔCt^ formula. The primers used were: *GAPDH* forward: 5′‐TGACTTCAACAGCGACACCCA‐3′; *GAPDH* reverse: 5′‐CACCCTGTTGCTGTAGCCAAA‐3′; *GPR81* forward: 5′‐TTCGTATTTGGTGGCAGGCA‐3′; *GPR81* reverse: 5′‐TTTCGAGGGGTCCAGGTACA‐3′; *MGMT* forward: 5’‐ACCGTTTGCGACTTGGTACTT‐3′; and *MGMT* reverse: 5’‐GGAGCTTTATTTCGTGCAGACC‐3′.

### 2.10 Western blot

After plasmid transfection, T98G and A172 cells were treated with TMZ at different concentrations for 48 h. Cells were then lysed in RIPA buffer‐contained protease inhibitor and phosphatase inhibitor (Roche). The primary antibodies against GPR81 (Abcam, #ab106942), MGMT (Proteintech, #17195‐1‐AP), cleaved‐Caspase 3 (CST, #9661), Caspase 3 (CST, #9662), and β‐actin (Sigma‐Aldrich, #A5441) were used according to the manufacturers' recommendations.

### 2.11 Flow cytometry assay

For cell apoptosis analysis, transfected T98G and A172 cells were treated with TMZ at different concentrations for 48 h. The cells were then washed with cold phosphate‐buffered saline (PBS, 4°C). Annexin V‐fluorescein isothiocyanate (FITC) and propidium iodide (PI) double staining were used to sort cells in early or late apoptotic phase.

### 2.12 Statistical analysis

Data normality was examined using Shapiro–Wilk (*n* ≤ 50) or Kolmogorov–Smirnov test (*n* > 50). Normally distributed and non‐normally distributed variables were analyzed using Student *t*‐test and Mann–Whitney U test. Correlation of normally distributed and non‐normally distributed data was assessed by Pearson and Spearman correlation test. Categorical data were tested using Chi‐square test. Survival data (e.g., OS, progression‐free survival [PFS]) were compared using Kaplan–Meier curves and log‐rank test. The prognostic correlation and independence of each variable were evaluated using univariate and multivariate Cox regression models. Results of each cohort or subgroup were combined using meta‐analysis, where the inverse‐variance approach was applied using either fixed‐ or random effect models based on the heterogeneity test, with *I* ^2^ ≥ 50% or *p*‐value ≤0.05 considered to be statistically significant. Hierarchical cluster analysis (HCA) was performed using R package *hclust*. The area under the curve (AUC) of the receiver operating characteristic (ROC) curve was done by R package *survivalROC*, to evaluate the prediction ability of the risk score models. All statistical analyses were done within SPSS statistics v19.0, GraphPad Prism v8.4.3 and R software v3.6.3, with two‐sided *p*‐value ≤0.05 considered to be statistically significant.

## 3 RESULTS

### 3.1 DNA methylation variations of a 64‐CpG panel were specifically associated with TMZ efficacy in G‐CIMP−/un*MGMT* GBMs

**FIGURE 2:**
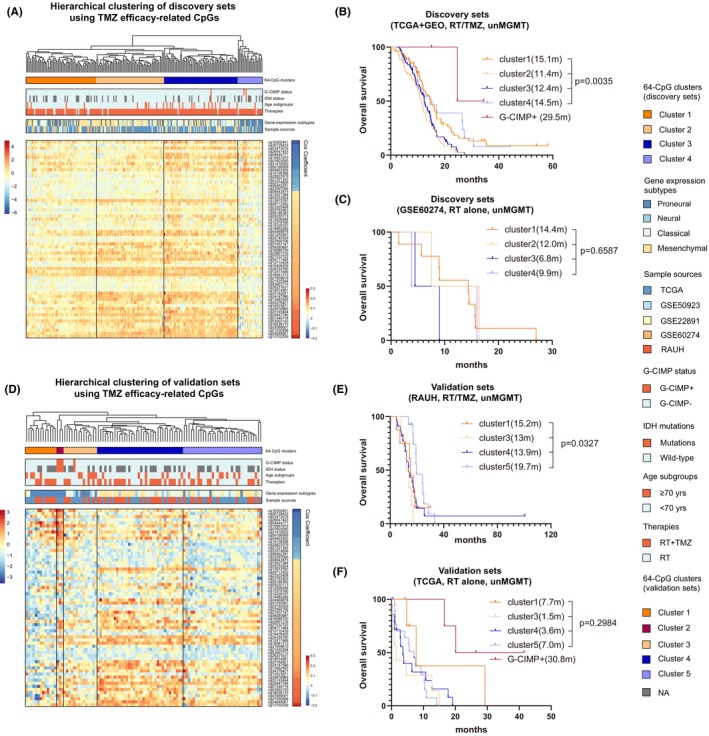
Identification and validation of CpG methylation with potential linkage to TMZ efficacy; the heatmaps of DNA methylation clusters defined by hierarchical clustering on 64 TMZ efficacy‐related CpGs (M‐values) in (A) discovery and (D) validation sets of un*MGMT* GBMs; each row represents a CpG; each column represents a sample which is grouped by hierarchical clustering; clinical and molecular features are indicated for each sample. Survival comparison among non‐G‐CIMP clusters in (B) discovery and (E) validation sets with combination of RT and TMZ. Survival comparison among non‐G‐CIMP clusters in (C) discovery and (F) validation sets with RT alone. Survival difference of each cluster was tested by log‐rank test with *p* value ≤0.05 as statistical significance. Hazard ratio (HR) and 95% confidence interval (CI) for survival curves were presented in Table S4.

Following the selection workflow (Figure 1B), we identified a panel of 64 TMZ efficacy‐related CpGs from discovery sets, each of which was significantly correlated with OS of G‐CIMP−/un*MGMT* GBMs treated with RT plus TMZ, rather than RT alone. In discovery sets, HCA on the 64‐CpG methylation data divided all G‐CIMP−/un*MGMT* GBMs into four clusters (Figure 2A). Survival analyses showed that OS of RT/TMZ‐treated G‐CIMP‐ GBMs significantly differed while that of RT‐treated tumors were undistinguished across the clusters (Figure 2B,C). Similarly, HCA also yielded four clusters of G‐CIMP−/un*MGMT* GBMs in validation sets and confirmed the distinct prognostic correlations with different treatments (RT/TMZ vs. RT alone; Figure 2D–F). Together the results indicated that the 64‐CpG panel may serve as an epigenetic biomarker pool that may provide predictive information on TMZ response in G‐CIMP−/un*MGMT* GBMs.

### 3.2 Identification of a ten‐CpG signature with potential linkage to TMZ efficacy in G‐CIMP−/un*MGMT* GBMs

To simplify the HCA‐based classification and develop a clinically applicable prediction model, we performed multivariate Cox regression analyses and reduced the 64‐CpG panel into a panel of 10 CpGs, each of which was supposed to provide significant, complementary, and independent prediction information for TMZ sensitivity in G‐CIMP−/unMGMT GBMs (Table 1). Accordingly, a RISK‐score formula was established as follows: risk score = (−0.313 × M‐value of cg26728422) + (−0.266 × M‐value of cg03473518) + (−0.240 × M‐value of cg26647453) + (−0.204 × M‐value of cg16302441) + (0.120 × M‐value of cg12578166) + (0.146 × M‐value of cg14329157) + (0.179 × M‐value of cg13702536) + (0.181 × M‐value of cg22783363) + (0.217 × M‐value of cg26221631) + (0.236 × M‐value of cg22861316).

**TABLE 1:** Genomic and clinical information of the 10 CpGs with potential linkage to TMZ efficacy.

Probe ID	Chromosome	Relevant gene symbol	Relation to gene regions	Relation to CpG Island	Multivariable Cox regression coefficients
cg26728422	16	UNKL	5'UTR	Shore	−0.313
cg03473518	13	GJB6	5'UTR	Shore	−0.266
cg26647453	4	C4orf17	5'UTR	Open sea	−0.24
cg16302441	2	POMC	TSS1500	Island	−0.204
cg12578166	11	KCNQ1	Body	Shore	0.12
cg14329157	2	WDR69	TSS200	Shore	0.146
cg13702536	12	GPR81	TSS1500	Open sea	0.179
cg22783363	8	TNFRSF10D	TSS200	Island	0.181
cg26221631	11	BARX2	1stExon	Island	0.217
cg22861316	5	FABP6	Body	Open sea	0.236

Abbreviations: TMZ, temozolomide; TSS, transcription start site; UTR, untranslated region.

**FIGURE 3:**
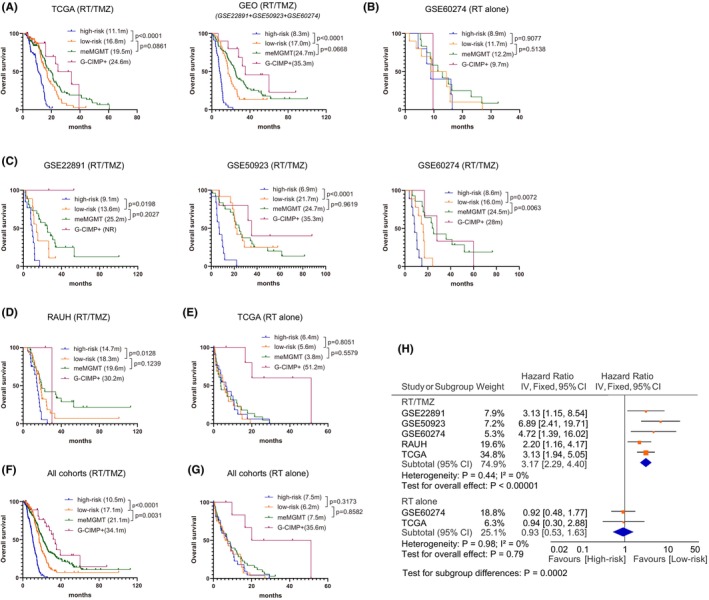
Prognostic performance of the 10‐CpG signature in G‐CIMP−/un*MGMT* GBMs in term of OS outcome; survival comparison among the subgroups characteristic of G‐CIMP+, meMGMT, G‐CIMP−/unMGMT low‐risk or G‐CIMP−/unMGMT high‐risk in (A) RT/TMZ‐treated and (B) RT‐treated discovery sets, as well as in (C) each GEO cohort; survival comparison among the above indicated subgroups in (D) RT/TMZ‐treated and (E) RT‐treated validation sets; pooled survival comparison using patient‐level data among the above indicated subgroups with (F) RT/TMZ or with (G) RT alone; (H) meta‐analysis using cohort‐level data between the risk subgroups with RT/TMZ or RT alone. Survival difference of each subgroup was tested by the log‐rank test with *p* value ≤0.05 as statistical significance. Hazard ratios [HR] from each dataset were combined by meta‐analysis, where the inverse‐variance approach was applied using either fixed‐ or random effect models based on the heterogeneity test, with *I* ^2^ ≥ 50% or *p* value ≤0.05 considered to be statistically significant. Hazard ratio (HR) and 95% confidence interval (CI) for survival curves were presented in Table S4.

Using the median RISK score (0.3028) in the RT/TMZ‐treated discovery sets as the cutoff, we divided all G‐CIMP−/un*MGMT* GBMs into low‐risk and high‐risk subgroups. In RT/TMZ‐treated TCGA cohort, low‐risk G‐CIMP−/un*MGMT* patients had longer OS than high‐risk patients (Figure 3A). Similarly, significant results were also observed in RT/TMZ‐treated GEO cohorts (GSE22891, GSE50923, and GSE60274 collectively; Figure 3A). The risk classification in each GEO cohort was also shown in Figure 3C. By contrast, in RT‐treated GSE60274 cohort, OS was not significantly different between the risk subgroups (Figure 3B). In addition, survival data of patients with G‐CIMP or me*MGMT* GBMs were shown for comparison (Figure 3).

**FIGURE 4:**
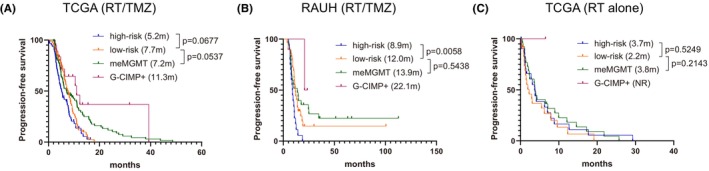
Prognostic performance of the 10‐CpG signature in G‐CIMP−/un*MGMT* GBMs in term of PFS outcome; survival comparison among the subgroups characteristic of G‐CIMP+, me*MGMT*, G‐CIMP−/un*MGMT* low‐risk or G‐CIMP−/un*MGMT* high‐risk in (A) RT/TMZ‐treated TCGA cohort, (B) RT/TMZ‐treated RAUH cohort, and (C) RT‐treated TCGA cohort. Survival difference of each subgroup was tested by log‐rank test with *p* value ≤0.05 as statistical significance. Hazard ratio (HR) and 95% confidence interval (CI) for survival curves were presented in Table S4.

To further evaluate the performance of the 10‐CpG signature, we applied the RISK‐score signature to validation sets with different treatments. Expectedly it accurately predicted OS in RT/TMZ‐treated RAUH cohort of G‐CIMP−/un*MGMT* GBMs but was not associated with OS in RT‐treated TCGA cohort (Figure 3D,E). Patient‐level and cohort‐level meta‐analyses both yielded distinct prognostic correlations of the 10‐CpG signature among different treatment subpopulations (Figure 3F–H). Similar results were observed in terms of PFS outcome (Figure 4). Cox regression analyses confirmed the 10‐CpG signature as a potent and independent survival predictor for G‐CIMP−/un*MGMT* GBMs treated with RT/TMZ, rather than RT alone (Table S1). The results indicated that, instead of a general prognostic factor regardless of treatment, the 10‐CpG signature may have a specific linkage to TMZ efficacy in G‐CIMP−/un*MGMT* GBMs.

### 3.3 The 10‐CpG signature may be a potent predictive factor aiding in TMZ decision‐making

**FIGURE 5:**
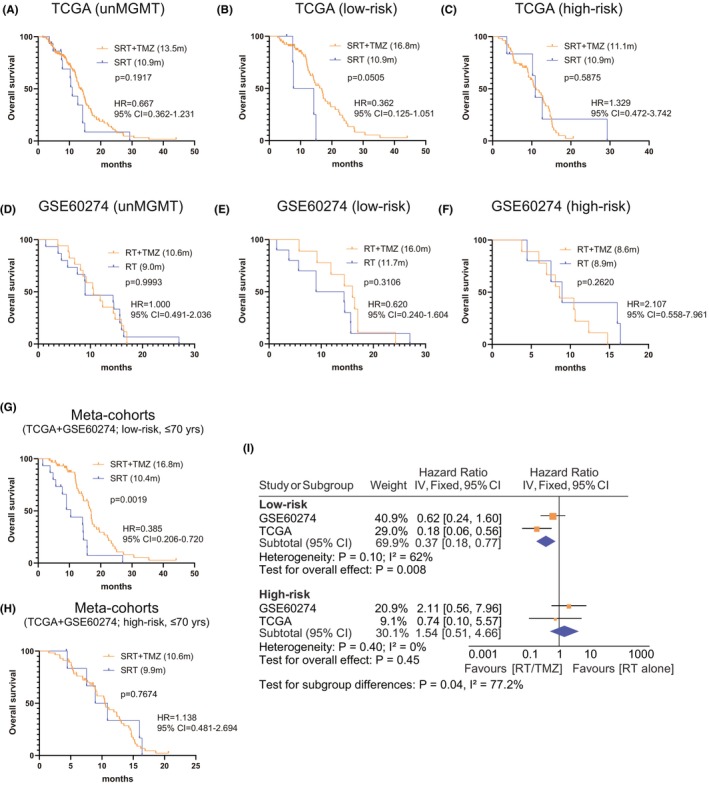
The predictive performance of the 10‐CpG signature in G‐CIMP−/unMGMT GBMs; Survival difference between different treatments (SRT/TMZ vs. SRT) in G‐CIMP−/unMGMT GBMs from (A) TCGA and (D) GSE60274 cohort; Interaction analysis between treatments (SRT/TMZ vs. SRT alone), and risk subgroups (low‐risk vs. high‐risk) in (B,C) TCGA and (E,F) GSE60274 cohort. Pooled survival comparisons using patient‐level data between different treatments (SRT/TMZ vs. SRT) in (G) low‐risk and (H) high‐risk subgroups; Meta‐analysis using cohort‐level data between different treatments (SRT/TMZ vs. SRT) in each risk subgroup incorporating only younger patients (≤70 years). Survival difference of each treatment subgroup was tested by the log‐rank test with *p* value ≤0.05 as statistical significance. Hazard ratios [HR] from each dataset were combined by meta‐analysis, where the inverse‐variance approach was applied using either fixed‐ or random effect models based on the heterogeneity test, with *I* ^2^ ≥ 50% or *p* value ≤0.05 considered to be statistically significant.

To account for potential bias of assigned treatment regimens, only patients receiving standard RT (SRT) with or without (concurrent or adjuvant) TMZ were included for the interaction analyses between different risk subgroups (low‐risk vs. high‐risk) and different treatments (SRT/TMZ vs. SRT). In line with previous report,^33^ G‐CIMP−/un*MGMT* GBMs appeared not to benefit much from SRT/TMZ as compared to SRT (Figure 5A,D). The interaction analyses showed that SRT/TMZ appeared to confer an OS benefit to low‐risk G‐CIMP−/un*MGMT* GBMs (Figure 5B,E) but was associated with similar OS in high‐risk patients (Figure 5C,F).

No significant difference was observed in patient baseline information (e.g., surgery, gender, or KPS) between subgroups with different treatment and different risk subgroups except for a high proportion of elderly patients (>70 years) in RT‐treated subgroups (data not shown). Patient‐level and cohort‐level meta‐analyses only incorporating younger patients (≤ 70 years) were thus performed, which yielded a statistically significant OS difference between SRT/TMZ‐treated and SRT‐treated subgroups (Figure 5G–I). Cox regression analyses confirmed SRT/TMZ as a better option for low‐risk patients, but not for high‐risk ones (Table S2). Together, the results indicated that the 10‐CpG signature may represent a promising predictive model for TMZ response and be helpful for selecting patients who are likely to benefit from the addition of TMZ.

### 3.4 Clinical and molecular correlations of the 10‐CpG signature

**FIGURE 6:**
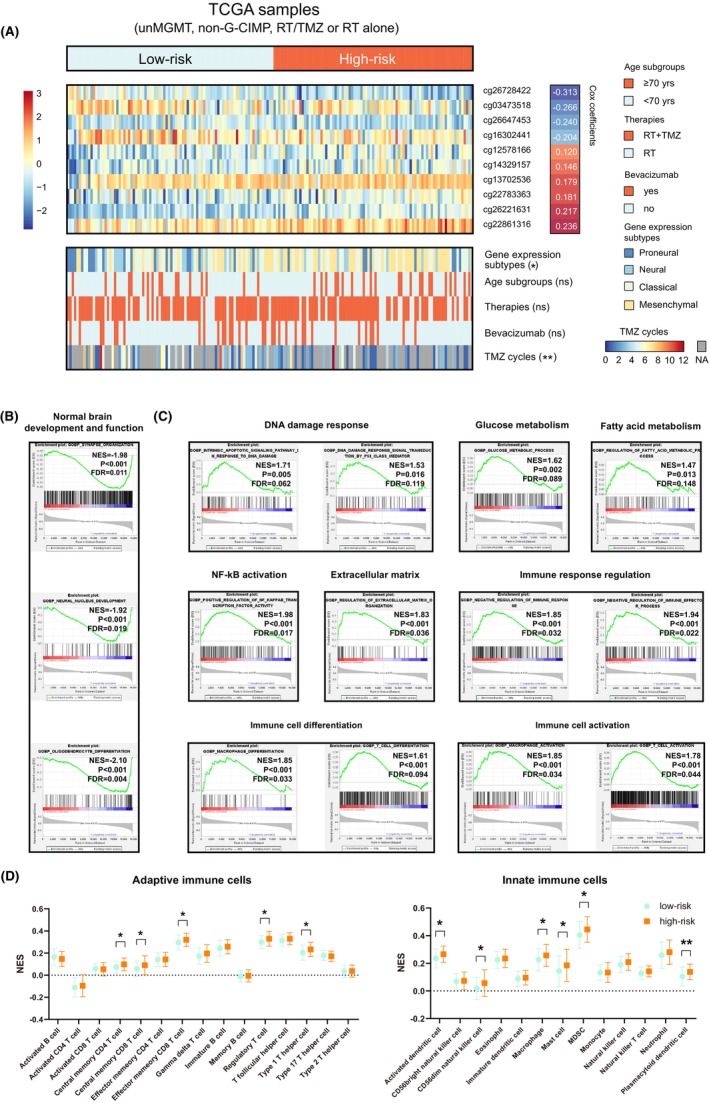
Molecular and biological correlations of the 10‐CpG signature using TCGA multi‐omics data; (A) heatmaps of the methylation levels (M‐values) of the 10 CpGs; each row represents a CpG and each column represents a sample which is ranked by its risk score. Clinical and molecular features are indicated for each sample, and multivariable Cox coefficients are indicated for each CpG; Representative GSEA enrichment plots of the highly enriched gene sets in (B) low‐risk tumors and in (C) high‐risk tumors; (D) the abundance of adaptive and innate immune infiltrating cells between low‐risk and high‐risk tumors. Categorical data (e.g., gene expression, age subgroup, therapies, and the use of bevacizumab) were tested by Chi‐square test. Data of TMZ cycles did not pass the normality test and were compared using Mann–Whitney U test. Data of Normalized enrichment scores (NESs) passed the normality test, and were compared using Student *t*‐test. Statistical significance was indicated at the level of ns >0.05, * < 0.05, ** <0.01, *** < 0.001 and **** < 0.0001. ns, non‐significant.

In TCGA G‐CIMP−/un*MGMT* GBMs, the 10‐CpG signature was found to be significantly associated with different gene expression subtypes; low‐risk tumors were enriched with proneural subtype, and high‐risk tumors were enriched with mesenchymal subtype (Figure 6A). In addition, the treatment cycles of TMZ were significantly higher in low‐risk tumors (median cycle: 4) than in high‐risk tumors (median cycle: 3; Figure 6A). GSEA showed that low‐risk tumors were enriched with gene sets related to normal brain development and function (Figure 6B; Table S3), while high‐risk tumors were enriched with a variety of cancer‐promoting signatures and especially those relevant to DNA damage response, glucose metabolism, fatty acid metabolism, NF‐kB activation, extracellular matrix (ECM) remodeling, immune response, and immune cell function (Figure 6C; Table S3). ssGSEA showed that high‐risk tumors were associated with a higher abundance of some TIIC types and particularly those immunosuppressive cells (e.g., regulatory T cells [Treg], myeloid‐derived suppressor cells [MDSCs]; Figure 6D). The results suggested that the enhanced TMZ resistance in high‐risk tumors may be attributable to a complex network of multiple tumorigenic or chemo‐resistant mechanisms, rather than a single molecular player.

### 3.5 *GPR81* may exhibit distinct impacts on TMZ resistance that depend on *MGMT* status

**FIGURE 7:**
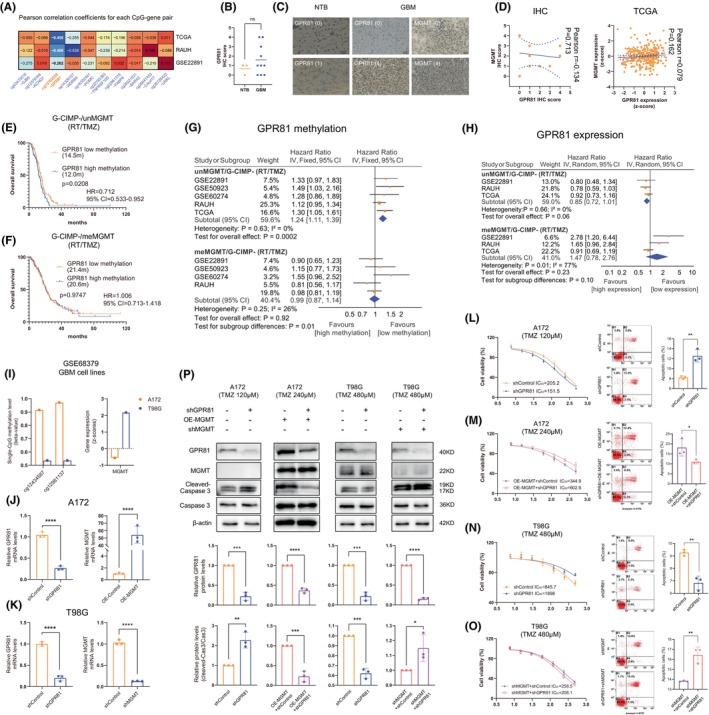
The impacts of *GPR81* on TMZ resistance of GBM cells that may depend on *MGMT* status; (A) Pearson correlation coefficients for each CpG‐gene pair from included cohorts; (B). The IHC scores of *GPR81* between NTB and GBM samples from Neurosurgery Department, Xijing Hospital; (C) Representative IHC images of *GPR81* and *MGMT* in NTB or GBM samples, with corresponding IHC scores; (D) Pearson correlation between *MGMT* and *GPR81* at protein and mRNA levels in local samples; (E,F) Survival difference between low versus high methylation of *GPR81* among RT/TMZ‐treated (E) G‐CIMP−/un*MGMT* and (F) G‐CIMP−/me*MGMT* GBMs; the median methylation value (M‐value: 2.0708) from RT/TMZ‐treated G‐CIMP−/un*MGMT* GBMs was used for stratifying low versus high methylation; (G,H) Meta‐analyses for (G) *GPR81* methylation‐based groups and (H) *GRP81* expression‐based groups in RT/TMZ‐treated non‐G‐CIMP GBMs with each *MGMT* methylation status; the median expression value (Z‐score: −0.1992) from RT/TMZ‐treated G‐CIMP−/un*MGMT* GBMs was used for stratifying low versus high expression. (I) Methylation and expression status of *MGMT* in A172 and T98G cells from GSE68379; (J) Validation of *GPR81* knockdown and *MGMT* overexpression in A172 cells by qRT‐PCR; (K) Validation of *GPR81* knockdown and *MGMT* knockdown in T98G cells by qRT‐PCR; (L) *GPR81* knockdown increased TMZ sensitivity and cell apoptosis to TMZ treatment in A172 cells originally with no detectable *MGMT* expression; (M) *GPR81* knockdown decreased TMZ sensitivity and cell apoptosis to TMZ treatment in *MGMT*‐overexpressed A172 cells; (N) *GPR81* knockdown decreased TMZ sensitivity and cell apoptosis to TMZ treatment in T98G cells originally expressing *MGMT*; (O) *GPR81* knockdown increased TMZ sensitivity and cell apoptosis to TMZ treatment in *MGMT*‐silenced T98G cells; (P) Western bolt results in A172 and T98G cells treated with TMZ; Hazard ratios [HR] from each dataset were combined by meta‐analysis, where the inverse‐variance approach was applied using either fixed‐ or random effect models based on the heterogeneity test, with *I* ^2^ ≥ 50% or *p* value ≤0.05 considered to be statistically significant. All continuous data passed normality test except for IHC scores. Statistical significance was indicated at the level of ns >0.05, * < 0.05, ** <0.01, *** < 0.001 and **** < 0.0001. ns, non‐significant.

The 10‐CpG methylation and the expression of their corresponding genes between NTBs and GBMs with each *MGMT* methylation status were presented in Figure S1. Pearson correlation analyses showed that only one CpG‐gene pair (cg13702536 and *GPR81*) showed stably and significantly negative correlations between CpG methylation and gene expression (Figure 7A). This CpG‐gene pair was thus selected for further analyses. *GPR81* mRNA and protein levels were not significantly different between NTBs and GBMs (Figure 7B,C and Figure S1). The expression of *GPR81* was also not significantly correlated with that of *MGMT* (Figure 7C,D). Interestingly, meta‐analyses showed that both *GPR81* methylation and expression appeared to be significantly associated with OS in un*MGMT* tumors, but not in me*MGMT* tumors when treated with RT/TMZ, indicting potential *MGMT*‐dependent impacts of *GPR81* on TMZ efficacy in G‐CIMP‐ GBMs.

To test this hypothesis in in vitro experiments, we selected two GBM cell lines (A172 and T98G) which were reported to have similarly high *GPR81* expression but distinct *MGMT* methylation and expression status in GSE68379^28^; specifically A172 was characteristic of high methylation and low expression of *MGMT*, while T98G was characteristic of low methylation and high expression of *MGMT* (Figure 7I and Figure S2). The expression of *GPR81* and *MGMT* was validated in our A172 and T98G cells (Figure S3), followed by validation of *GPR81* knockdown (Figure 7J,K). We found that *GPR81* knockdown significantly increased TMZ sensitivity and cell apoptosis when exposed to TMZ in *MGMT*‐deficient A172 cells (Figure 7L,P). However in *MGMT*‐overexpressed A172 cells, *GPR81* knockdown conversely decreased TMZ sensitivity and TMZ‐treated apoptosis (Figure 7M,P). Moreover, in T98G cells originally expressing high level of *MGMT*, knockdown of *GPR81* did decrease TMZ efficacy (Figure 7N,P) while in *MGMT*‐silenced T98G cells, *GPR81* knockdown conversely increased TMZ sensitivity (Figure 7O,P and Figure S4). The results together indicated that the contributions of *GPR81* to TMZ resistance may depend on *MGMT* expression status in GBMs; briefly, *GPR81* may enhance TMZ sensitivity when GBM cells highly expressed *MGMT*, while *GPR81* may enhance TMZ resistance when *MGMT* expression was absent or largely repressed in GBM cells.

## 4 DISCUSSION

TMZ has long been recognized as the first‐choice chemotherapy for treating primary GBMs.^1^, ^2^ Unfortunately, it cannot benefit every GBM patients and many are resistant to this genotoxic drug.^2^ Molecular biomarkers have been increasingly reported for aiding TMZ choice, among which *MGMT* methylation status stands for the most widely validated predictive biomarker.^1^, ^2^ However, *MGMT* methylation had limited use in guiding TMZ in clinical practice due to lack of a straightforward relationship between its detection and TMZ choice in GBMs.^3^ Although TMZ yielded much reduced benefits to un*MGMT* tumors as compared to me*MGMT* tumors, it is unlikely to withdraw from standard treatment, since there is lack of effective alternative therapies, and TMZ still benefits for some un*MGMT* cases.^3^ However, it should be noted that TMZ is not a cost‐effective anti‐GBM therapy, and its overuse can result in overconsumption of health resources, raise medical cost to caregivers, and increase risk of drug toxicity.^34^ Therefore, identifying potent predictive biomarkers, other than *MGMT* methylation, that can be useful for selecting subgroups of un*MGMT* patients with good sensitivity to TMZ, may represent a promising approach for optimizing decision‐making on TMZ.

DNA methylation represented ideal biomarker candidates for precision oncology.^35^ The mainstream expression‐ (e.g., RNA, protein) based biomarkers have critical weaknesses as their information can be unstable and even misleading owing to the high dynamic metabolism of RNA and protein, and the instable physical–chemical structure when stored in biological specimens.^35^, ^36^, ^37^ The genetic (e.g., mutations, copy number variation)‐based biomarkers also have clinical drawbacks such as inability to distinguish non‐tumor cell contamination and tumor cells of origin, and disallowance for a quantitative detection.^35^, ^36^, ^37^ By contrast, cancer‐specific DNA methylation alterations can be stable over time and easy to get and store, and usually carried abundant biological information, and occurred at the very early phase of carcinogenesis preceding other molecular alterations.^35^, ^36^, ^37^ The last and most appealing advantage is the availability of epigenetic drugs that could reverse aberrant DNA methylation modifications, making it not only an indicator of certain features of a given cancer but also a druggable target to cure the disease.^38^

In this study, by integrating epigenome data, survival outcome, and treatment information of multi‐sourced GBM cohorts, we identified a panel of 64 CpGs that may be specifically linked to TMZ efficacy in G‐CIMP−/un*MGMT* GBMs. To construct a clinically applicable prediction model, we employed a multi‐step selection workflow to screen out an optimal combination of a few number of CpGs, each of which not only conferred potent and independent prediction ability but also coordinated with and complemented each other. Finally, a 10‐CpG panel was identified and combined using a RISK‐score model. Testing the 10‐CpG signature in different cohorts of G‐CIMP−/un*MGMT* GBMs showed that the defined low‐risk tumors were stably associated with better OS than high‐risk tumors when treated with RT/TMZ but not RT alone. So, it is inferred that the risk signature may be informative of distinct TMZ efficacy in G‐CIMP−/un*MGMT* GBMs, instead of a treatment‐independent prognostic biomarker.^6^ Furthermore, the interaction analyses revealed that, as compared to RT alone, RT/TMZ was more beneficial to low‐risk patients but yielded similar OS outcomes in high‐risk patients. These results indicated that the 10‐CpG risk signature may serve as a promising predictive factor for TMZ efficacy in G‐CIMP−/un*MGMT* GBMs and may be helpful for providing predictive information on the likely response to TMZ and identifying appropriate patients who are most likely to benefit from TMZ.^6^

Sparse studies have been focused on discovering prognostic or predictive factors for G‐CIMP−/unMGMT GBMs, and a few prediction models been reported with potential clinical value.^39^, ^40^, ^41^ Like our study, Chai et al.^39^ reported a 31‐CpGs risk signature that predicted survival of TMZ‐treated un*MGMT* GBMs. Ye et al.^40^ reported a prognostic 13‐gene risk signature that was validated in four RT/TMZ‐treated cohorts of *IDH* wild‐type (wt) and un*MGMT* GBMs. Li et al.^41^ proposed a 6‐lncRNA immune‐relevant risk signature that predicted survival in *IDH*wt/un*MGMT* GBMs. Table 2 compares the published signatures with our risk signature. The 10‐CpG signature appeared to have a good predictive ability than the published models, with the highest AUC values at 1 year, 2 years, and 3 years in TCGA samples (Table 2). Also the present study may have advantages in the following aspects. First, abundant sample sources with relatively large sample size were used for discovery and validation of the risk model. Second, the treatment information was incorporated into the CpG selection, which is a key variable to distinguish a predictive factor from a prognostic one.^6^ Third, the predictive value of the risk signature in our study was observed with a prospective objective on building a predictive model for TMZ response, instead of a spurious finding from a post‐hoc subgroup analysis. Finally, interaction analyses were performed to compare the survival benefits of different treatment regimens in each risk subgroup, which could provide a direct guide on TMZ usage in specific subpopulations.

**TABLE 2:** Comparison of our 10‐CpG signature with published signatures for un*MGMT* GBMs.

Author [ref]	Year	Signature	Molecular type	Biomarker type	Treatment correlation	Sample source	Interaction analysis between treatment and signature	AUC at each time point^a^		
								1‐year	2‐years	3‐years
Chai^32^	2019	31‐CpG risk scores	DNA methylation	Prognostic	TMZ‐related	Discovery set: TCGA Validation set: TCGA	Yes	0.632	0.806	0.646
Ye^33^	2019	13‐gene risk scores	Expression	Prognostic	RT/TMZ‐related	Discovery set: TCGA; Validation set: TCGA; GSE4271; CGGA; Xiangya cohort	No	0.659	0.774	0.522
Li^34^	2020	6‐lncRNA risk scores	Expression	Prognostic	Unclear	Discovery set: TCGA; CGGA Validation set: CGGA	No	0.528	0.362	—
Present study		10‐CpG risk scores	DNA methylation	Predictive	RT/TMZ‐related; RT‐unrelated	Discovery set: TCGA; GSE22891; GSE60274; GSE50923; Validation set: RAUH, TCGA	Yes	0.662	0.876	0.827

Abbreviations: AUC, area under the curve; CGGA, China Glioma Genome Atlas; lncRNA, long non‐coding RNA; MGMT, O‐6‐methylguanine‐DNA methyltransferase; RAUH, Rennes and Angers University Hospitals; Ref, reference; RT, radiotherapy; TCGA, the Cancer Genome Atlas; TMZ, temozolomide.

^a^ AUC of each signature was assessed in RT/TMZ‐treated G‐CIMP−/unMGMT GBMs from TCGA.

The biological implications of the 10‐CpG signature may provide molecular clues behind its predictive ability for TMZ response. As highlighted by previous studies^42^ that a complex and intertwined network of multiple molecular mechanisms may together determine the therapeutic resistance of GBMs, our bioinformatic analyses showed that the enhanced TMZ resistance observed in high‐risk tumors may be partially attributable to the high enrichment of various cancer‐promoting or therapy‐resistant signatures involving in DNA damage response, energy metabolism, NF‐kB activation, ECM remodeling, and tumor immunity, as well as an increased abundance of immunosuppressive cells (e.g., Treg and MDSCs). In another word, a prediction model incorporating multiple variables that are indicative of different aspects of TMZ efficacy‐related molecular features, like our risk signature, may be more informative of chemo‐resistance in G‐CIMP−/un*MGMT* GBMs, instead of a single‐marker model.

DNA methylation represents one critical layer of control in gene expression.^35^, ^37^, ^38^ It is reasonable to assume that the multi‐CpG signature may contribute to TMZ resistance via regulating the expression of relevant genes. In our signature, only one CpG‐gene pair (cg13702536 and *GPR81*) was found to show stable and significant correlation between DNA methylation and gene expression across different datasets, suggesting that *GPR81* may be epigenetically controlled by DNA methylation. *GPR81* has been reported to have multifunctional roles in promoting malignant behaviors of tumor cells by regulating energy metabolism,^43^ angiogenesis,^44^ therapeutic resistance,^45^ and tumor immunity.^46^, ^47^ Surprisingly, in in vitro GBM cell experiments, we have revealed the potential *MGMT*‐dependent impacts of *GPR81* on TMZ resistance; specifically in GBM cells with high methylation (or low expression) of *MGMT*, e.g., A172‐ and *MGMT*‐silenced T98G cells, *GPR81* may enhance TMZ resistance while in GBM cells with low methylation (or high expression) of *MGMT*, e.g., MGMT‐overexpressed A172 and T98G cells, *GPR81* may increase TMZ sensitivity. In line with experimental data, survival analyses also supported the distinct predictive abilities of *GPR81* expression (or methylation) in RT/TMZ‐treated G‐CIMP‐ GBMs with different *MGMT* methylation statues. However, the absence of apparent predictive ability in me*MGMT* tumors indicated that the tumor intrinsic *GPR81* expression may not act as a major contributor to TMZ resistance among the complex molecular mechanisms conferred by the entire tumor microenvironments in me*MGMT* samples. By contrast, tumor intrinsic *GRP81* expression may be a dominant player for TMZ efficacy among the un*MGMT* GBM microenvironments as supported by the significant predictive ability in clinical samples and the significant impacts on TMZ resistance in GBM cell lines. In summary, the clinical and experimental data of *GPR81* may provide additional layer of evidence supporting the predictive ability of the 10‐CpG signature in G‐CIMP−/unMGMT GBMs. Moreover, the *MGMT*‐dependent roles of *GPR81* highlighted the complexity and sophistication of the underlying molecular mechanisms that eventually define the resistant nature of GBMs. However, by far, the data are too preliminary to draw conclusion. Future studies are needed to address how *MGMT* affects the functions of *GPR81* in TMZ resistance of GBMs, and what *MGMT* status triggers the function transition of *GPR81*.

Functional reports on the other CpG‐relevant genes may also provide biological clues for the predictive ability of our risk signature. *GJB6* (harboring cg03473518) encodes a tumor‐suppressive gap junction protein and may prevent GBM growth via rewiring glucose metabolism and inhibiting stemness.^48^, ^49^ *KCNQ1* (harboring cg12578166) encodes a voltage‐dependent K+ channel and acts both as a target gene and regulator of the Wnt/β‐catenin pathway.^50^ Loss of *KCNQ1* has been reported to exert anti‐tumor functions via promoting epithelial‐to‐mesenchymal transition (EMT) and disrupting adheren junctions in epithelial cancers.^50^ *WDR69* (harboring cg14329157), also called dynein assembly factor with WD repeats 1 (DAW1), belongs to the WD‐repeat domain (WDR) family and plays vital roles in cilia motility.^51^ *WDR69* hypermethylation was found to be associated with unfavorable prognosis in hepatocellular carcinoma.^52^ *TNFRSF10D* (harboring cg22783363) encodes a plasma membrane‐located TNF‐related apoptosis‐inducing ligand (TRAIL) decoy receptor, and negatively regulates TRAIL‐induced apoptosis.^53^ Hypermethylation and silencing of *TNFRSF10D* have been reported to occur in multiple cancer types and be associated with poor survival and resistance to DNA‐damaging drugs.^53^, ^54^ *FABP6* (harboring cg22861316), encoding a bile acid‐binding protein, is physiologically involved in fatty acids metabolism.^55^ Recently, dysregulation and dysfunction of *FABP6* have been reported to be involved in multiple cancers including GBMs.^55^, ^56^, ^57^ In GBM cells, FABP6 inhibition reversed the malignant phenotypes of tumor cells and increased TMZ sensitivity.^55^ *BARX2* (harboring cg26221631) encodes a member of the homeobox transcription factor family that controls cell adhesion and cytoskeleton remodeling.^58^ Downregulation of *BARX2*, partially by CGI hypermethylation, has been reported to correlate with enhanced aerobic glycolysis and aggressive behaviors of tumor cells and be indicative of poor prognosis.^59^ *POMC* (harboring cg16302441) encodes a pro‐hormone that gives rise to various active peptides such as adrenocorticotropic hormone (ACTH) and melanocyte stimulating hormones (MSHs).^60^ Some *POMC*‐derived peptides have been reported to have vital roles in neuroendocrine tumors such as guiding optimal choice of chemotherapy.^60^ By far, little is known about the relevance of *C4orf17* (harboring cg26647453) and *UNKL* (harboring cg26728422) in cancers. The multi‐CpG signature may unlikely impact TMZ resistance via direct transcriptional control of the above genes as no significant correlations were observed for the nine CpG‐gene pairs. However, in addition to classical epigenetic regulation mechanism, DNA methylation abnormalities may have broader biological effects by affecting heterochromatin structures, leading to loss of epigenetic regulation and resulting in hypervariability of gene expression.^36^, ^37^, ^61^ Future studies are needed to explore the molecular machinery on TMZ resistance behind the CpG members that may not directly control local gene expression.

Limitations exist in the present study. Our finding should be carefully interpreted due to the following shortcomings, such as lack of validation in a randomized setting or in a prospective manner, potential patient selection bias in retrospectively collected cohorts, very few samples for RT‐treated patients, heterogeneous treatment regimens, and incomplete clinical data. Moreover, the risk signature was built on high‐throughput DNA methylation detection platform, which is not clinically available and not economical for routine testing. Therefore, the risk signature in its current form is not ready for daily use and should be modified and validated by a more common detection system, such as pyrosequencing.^47^ Finally, the histochemical characterization of glioma in our study is technically conventional and has limited scope. New histo‐methodology, such as tissue clearing and quantitative ultramicroscopy, may provide more comprehensive molecule‐histology information.^62^

In conclusion, we firstly reported a panel of 64 TMZ efficacy‐related CpGs and then built a 10‐CpG‐based RISK‐score signature that may robustly and stably predict response to TMZ in G‐CIMP−/un*MGMT* GBMs, a subtype characteristic of high TMZ resistance. Experimental data revealed potential *MGMT*‐dependent roles of *GPR81* in TMZ resistance, highlighting the complexity of the chemo‐resistant mechanisms in GBMs. The 10‐CpG signature may be helpful for guiding TMZ choice in such subpopulation. Future studies are needed to explore the molecular mechanisms underlying the risk signature and to translate it into routine practice.

## AUTHOR CONTRIBUTIONS

Conception and design of the study: BBL, YHW, FFL, AAY, and SQZ. Provision of study material or patients: JJH, SL, GCM, ZJZ, YLH, and AE. Acquisition and assembly of data: BBL, YHW, JJH, WJY, CYS, and HZW. Project administration, Software, methodology: BBL, YHW, FFL, and AAY. Analysis and interpretation of results: BBL, YHW, FFL, HFK, AAY, and SQZ. Manuscript writing: All authors. Final approval of manuscript: All authors.

## FUNDING INFORMATION

This work was partially funded by grants from National Natural Science Foundation of China (No. 81402049, 81802486), Shaanxi Province Natural Science Foundation (No.2023‐JCYB‐641), Shandong Province Natural Science Foundation (No.ZR2020QH0233), and by Grants from the Brittany Region (France) et the FEDER (Europe).

## CONFLICT OF INTEREST STATEMENT

The authors have no conflict of interest.

## Supporting information


Figure S1.

Figure S2.

Figure S3.

Figure S4.

Table S1.

Table S2.

Table S3.

Table S4.

Data S1.

## Data Availability

The datasets used and/or analyzed during the current study are available from the corresponding author on reasonable request or public databases; TCGA: https://tcga‐data.nci.nih.gov; GEO: https://www.ncbi.nlm.nih.gov/geo/.

## References

[1] Tan AC , Ashley DM , Lopez GY , Malinzak M , Friedman HS , Khasraw M . Management of glioblastoma: state of the art and future directions. CA Cancer J Clin. 2020;70:299‐312. 32478924 10.3322/caac.21613

[2] Tomar MS , Kumar A , Srivastava C , Shrivastava A . Elucidating the mechanisms of Temozolomide resistance in gliomas and the strategies to overcome the resistance. Biochim Biophys Acta Rev Cancer. 2021;1876:188616. 34419533 10.1016/j.bbcan.2021.188616

[3] Yin AA , Zhang LH , Cheng JX , et al. Radiotherapy plus concurrent or sequential temozolomide for glioblastoma in the elderly: a meta‐analysis. PloS One. 2013;8:e74242. 24086323 10.1371/journal.pone.0074242 PMC3782499

[4] Brandner S , McAleenan A , Kelly C , et al. MGMT promoter methylation testing to predict overall survival in people with glioblastoma treated with temozolomide: a comprehensive meta‐analysis based on a Cochrane Systematic Review. Neuro Oncol. 2021;23:1457‐1469. 34467991 10.1093/neuonc/noab105 PMC8408882

[5] Hu YH , Jiao BH , Wang CY , Wu JL . Regulation of temozolomide resistance in glioma cells via the RIP2/NF‐kappaB/MGMT pathway. CNS Neurosci Ther. 2021;27:552‐563. 33460245 10.1111/cns.13591 PMC8025621

[6] Yin AA , Zhang LH , Cheng JX , et al. The predictive but not prognostic value of MGMT promoter methylation status in elderly glioblastoma patients: a meta‐analysis. PloS One. 2014;9:e85102. 24454798 10.1371/journal.pone.0085102 PMC3890309

[7] Yin AA , He YL , Etcheverry A , et al. Novel predictive epigenetic signature for temozolomide in non‐G‐CIMP glioblastomas. Clin Epigenetics. 2019;11:76. 31088577 10.1186/s13148-019-0670-9 PMC6515684

[8] Li B , Wang J , Liu F , et al. A novel pseudogene methylation signature to predict temozolomide outcome in non‐G‐CIMP glioblastomas. J Oncol. 2022;2022:6345160. 35712126 10.1155/2022/6345160 PMC9194959

[9] Roychowdhury S , Chinnaiyan AM . Translating cancer genomes and transcriptomes for precision oncology. CA Cancer J Clin. 2016;66:75‐88. 26528881 10.3322/caac.21329 PMC4713245

[10] Liu D , Yang T , Ma W , Wang Y . Clinical strategies to manage adult glioblastoma patients without MGMT hypermethylation. J Cancer. 2022;13:354‐363. 34976195 10.7150/jca.63595 PMC8692679

[11] Malta TM , de Souza CF , Sabedot TS , et al. Glioma CpG Island methylator phenotype (G‐CIMP): biological and clinical implications. Neuro Oncol. 2018;20:608‐620. 29036500 10.1093/neuonc/nox183 PMC5892155

[12] Lu Y , Kwintkiewicz J , Liu Y , et al. Chemosensitivity of IDH1‐mutated gliomas due to an impairment in PARP1‐mediated DNA repair. Cancer Res. 2017;77:1709‐1718. 28202508 10.1158/0008-5472.CAN-16-2773 PMC5380481

[13] Li S , Chou AP , Chen W , et al. Overexpression of isocitrate dehydrogenase mutant proteins renders glioma cells more sensitive to radiation. Neuro Oncol. 2013;15:57‐68. 23115158 10.1093/neuonc/nos261 PMC3534418

[14] Chai R , Li G , Liu Y , et al. Predictive value of MGMT promoter methylation on the survival of TMZ treated IDH‐mutant glioblastoma. Cancer Biol Med. 2021;18:272‐282. 33628600 10.20892/j.issn.2095-3941.2020.0179 PMC7877176

[15] Radke J , Koch A , Pritsch F , et al. Predictive MGMT status in a homogeneous cohort of IDH wildtype glioblastoma patients. Acta Neuropathol Commun. 2019;7:89. 31167648 10.1186/s40478-019-0745-z PMC6549362

[16] Chai R , Fang S , Pang B , et al. Molecular pathology and clinical implications of diffuse glioma. Chin Med J (Engl). 2022;135:2914‐2925. 36728558 10.1097/CM9.0000000000002446 PMC10106158

[17] Chai RC , Liu YQ , Zhang KN , et al. A novel analytical model of MGMT methylation pyrosequencing offers improved predictive performance in patients with gliomas. Mod Pathol. 2019;32:4‐15. 30291347 10.1038/s41379-018-0143-2

[18] Zhang K , Liu X , Li G , et al. Clinical management and survival outcomes of patients with different molecular subtypes of diffuse gliomas in China (2011‐2017): a multicenter retrospective study from CGGA. Cancer Biol Med. 2022;19:1460‐1476. 36350010 10.20892/j.issn.2095-3941.2022.0469 PMC9630520

[19] Noushmehr H , Weisenberger DJ , Diefes K , et al. Identification of a CpG Island methylator phenotype that defines a distinct subgroup of glioma. Cancer Cell. 2010;17:510‐522. 20399149 10.1016/j.ccr.2010.03.017 PMC2872684

[20] Bady P , Sciuscio D , Diserens AC , et al. MGMT methylation analysis of glioblastoma on the Infinium methylation BeadChip identifies two distinct CpG regions associated with gene silencing and outcome, yielding a prediction model for comparisons across datasets, tumor grades, and CIMP‐status. Acta Neuropathol. 2012;124:547‐560. 22810491 10.1007/s00401-012-1016-2 PMC3444709

[21] Du P , Zhang X , Huang CC , et al. Comparison of Beta‐value and M‐value methods for quantifying methylation levels by microarray analysis. BMC Bioinformatics. 2010;11:587. 21118553 10.1186/1471-2105-11-587 PMC3012676

[22] Brennan CW , Verhaak RG , McKenna A , et al. The somatic genomic landscape of glioblastoma. Cell. 2013;155:462‐477. 24120142 10.1016/j.cell.2013.09.034 PMC3910500

[23] Etcheverry A , Aubry M , de Tayrac M , et al. DNA methylation in glioblastoma: impact on gene expression and clinical outcome. BMC Genomics. 2010;11:701. 21156036 10.1186/1471-2164-11-701 PMC3018478

[24] Lai RK , Chen Y , Guan X , et al. Genome‐wide methylation analyses in glioblastoma multiforme. PloS One. 2014;9:e89376. 24586730 10.1371/journal.pone.0089376 PMC3931727

[25] Kurscheid S , Bady P , Sciuscio D , et al. Chromosome 7 gain and DNA hypermethylation at the HOXA10 locus are associated with expression of a stem cell related HOX‐signature in glioblastoma. Genome Biol. 2015;16:16. 25622821 10.1186/s13059-015-0583-7 PMC4342872

[26] Yin A , Shang Z , Etcheverry A , et al. Integrative analysis identifies an immune‐relevant epigenetic signature for prognostication of non‐G‐CIMP glioblastomas. Onco Targets Ther. 2021;10:1902071. 10.1080/2162402X.2021.1902071 PMC8018210 33854822

[27] Horvath S , Garagnani P , Bacalini MG , et al. Accelerated epigenetic aging in Down syndrome. Aging Cell. 2015;14:491‐495. 25678027 10.1111/acel.12325 PMC4406678

[28] Iorio F , Knijnenburg TA , Vis DJ , et al. A landscape of pharmacogenomic interactions in cancer. Cell. 2016;166:740‐754. 27397505 10.1016/j.cell.2016.06.017 PMC4967469

[29] Wang J , Zhang M , Liu YF , et al. Potent predictive CpG signature for temozolomide response in non‐glioma‐CpG Island methylator phenotype glioblastomas with methylated MGMT promoter. Epigenomics‐UK. 2022;14:1233‐1247. 10.2217/epi-2022-0344 36444681

[30] Johnson WE , Li C , Rabinovic A . Adjusting batch effects in microarray expression data using empirical Bayes methods. Biostatistics. 2007;8:118‐127. 16632515 10.1093/biostatistics/kxj037

[31] Subramanian A , Tamayo P , Mootha VK , et al. Gene set enrichment analysis: a knowledge‐based approach for interpreting genome‐wide expression profiles. Proc Natl Acad Sci U S A. 2005;102:15545‐15550. 16199517 10.1073/pnas.0506580102 PMC1239896

[32] Charoentong P , Finotello F , Angelova M , et al. Pan‐cancer immunogenomic analyses reveal genotype‐immunophenotype relationships and predictors of response to checkpoint blockade. Cell Rep. 2017;18:248‐262. 28052254 10.1016/j.celrep.2016.12.019

[33] Laperriere N , Weller M , Stupp R , et al. Optimal management of elderly patients with glioblastoma. Cancer Treat Rev. 2013;39:350‐357. 22722053 10.1016/j.ctrv.2012.05.008

[34] Wu B , Miao Y , Bai Y , et al. Subgroup economic analysis for glioblastoma in a health resource‐limited setting. PloS One. 2012;7:e34588. 22511951 10.1371/journal.pone.0034588 PMC3325281

[35] Galbraith K , Snuderl M . DNA methylation as a diagnostic tool. Acta Neuropathol Commun. 2022;10:71. 35527288 10.1186/s40478-022-01371-2 PMC9080136

[36] Yin AA , Lu N , Etcheverry A , et al. A novel prognostic six‐CpG signature in glioblastomas. CNS Neurosci Ther. 2018;24:167‐177. 29350455 10.1111/cns.12786 PMC6489960

[37] Gusyatiner O , Hegi ME . Glioma epigenetics: from subclassification to novel treatment options. Semin Cancer Biol. 2018;51:50‐58. 29170066 10.1016/j.semcancer.2017.11.010

[38] Issa JP . DNA methylation as a clinical marker in oncology. J Clin Oncol. 2012;30:2566‐2568. 22564986 10.1200/JCO.2012.42.1016

[39] Chai RC , Chang YZ , Wang QW , et al. A novel DNA methylation‐based signature can predict the responses of MGMT promoter unmethylated glioblastomas to temozolomide. Front Genet. 2019;10:910. 31611911 10.3389/fgene.2019.00910 PMC6776832

[40] Ye N , Jiang N , Feng C , et al. Combined therapy sensitivity index based on a 13‐gene signature predicts prognosis for IDH wild‐type and MGMT promoter unmethylated glioblastoma patients. J Cancer. 2019;10:5536‐5548. 31632497 10.7150/jca.30614 PMC6775685

[41] Li X , Meng Y . Immune‐related lncRNA risk signatures predict survival of IDH wild‐type and MGMT promoter unmethylated glioblastoma. Biomed Res Int. 2020;2020:1971284. 32851059 10.1155/2020/1971284 PMC7441444

[42] Rao V , Kumar G , Vibhavari R , et al. Temozolomide resistance: a multifarious review on mechanisms beyond O6MethylguanineDNA methyltransferase. CNS Neurol Disord Drug Targets. 2022;22:817‐831. 10.2174/1871527321666220404180944 35379142

[43] Ishihara S , Hata K , Hirose K , et al. The lactate sensor GPR81 regulates glycolysis and tumor growth of breast cancer. Sci Rep. 2022;12:6261. 35428832 10.1038/s41598-022-10143-w PMC9012857

[44] Lee YJ , Shin KJ , Park SA , et al. G‐protein‐coupled receptor 81 promotes a malignant phenotype in breast cancer through angiogenic factor secretion. Oncotarget. 2016;7:70898‐70911. 27765922 10.18632/oncotarget.12286 PMC5342597

[45] Soni VK , Shukla D , Kumar A , Vishvakarma NK . Curcumin circumvent lactate‐induced chemoresistance in hepatic cancer cells through modulation of hydroxycarboxylic acid receptor‐1. Int J Biochem Cell Biol. 2020;123:105752. 32325281 10.1016/j.biocel.2020.105752

[46] Brown TP , Bhattacharjee P , Ramachandran S , et al. The lactate receptor GPR81 promotes breast cancer growth via a paracrine mechanism involving antigen‐presenting cells in the tumor microenvironment. Oncogene. 2020;39:3292‐3304. 32071396 10.1038/s41388-020-1216-5

[47] Feng J , Yang H , Zhang Y , et al. Tumor cell‐derived lactate induces TAZ‐dependent upregulation of PD‐L1 through GPR81 in human lung cancer cells. Oncogene. 2017;36:5829‐5839. 28604752 10.1038/onc.2017.188

[48] Arun S , Ravisankar S , Vanisree AJ . Implication of connexin30 on the stemness of glioma: connexin30 reverses the malignant phenotype of glioma by modulating IGF‐1R, CD133 and cMyc. J Neurooncol. 2017;135:473‐485. 28875331 10.1007/s11060-017-2608-4

[49] Jothi J , Janardhanam VA , Rama K . Connexin 30 mediated rewiring of glucose metabolism in rat C6 xenograft and grades of glioma. Mol Cell Biochem. 2020;470:157‐164. 32462383 10.1007/s11010-020-03757-z

[50] Rapetti‐Mauss R , Berenguier C , Allegrini B , Soriani O . Interplay between ion channels and the Wnt/beta‐catenin signaling pathway in cancers. Front Pharmacol. 2020;11:525020. 33117152 10.3389/fphar.2020.525020 PMC7552962

[51] Bearce EA , Irons ZH , Craig SB , et al. Daw1 regulates the timely onset of cilia motility during development. Development. 2022;149: dev200017. 35708608 10.1242/dev.200017 PMC9270974

[52] Hao XY , Li AQ , Shi H , et al. A novel DNA methylation‐based model that effectively predicts prognosis in hepatocellular carcinoma. Biosci Rep. 2021;41:BSR20203945. 33634306 10.1042/BSR20203945 PMC7955104

[53] Narayan G , Xie D , Ishdorj G , et al. Epigenetic inactivation of TRAIL decoy receptors at 8p12‐21.3 commonly deleted region confers sensitivity to Apo2L/trail‐Cisplatin combination therapy in cervical cancer. Genes Chromosomes Cancer. 2016;55:177‐189. 26542757 10.1002/gcc.22325

[54] Ratzinger G , Mitteregger S , Wolf B , et al. Association of TNFRSF10D DNA‐methylation with the survival of melanoma patients. Int J Mol Sci. 2014;15:11984‐11995. 25003639 10.3390/ijms150711984 PMC4139825

[55] Pai FC , Huang HW , Tsai YL , et al. Inhibition of FABP6 reduces tumor cell invasion and angiogenesis through the decrease in MMP‐2 and VEGF in human glioblastoma cells. Cells‐Basel. 2021;10:2782. 10.3390/cells10102782 PMC8534568 34685761

[56] Lin CH , Chang HH , Lai CR , et al. Fatty acid binding protein 6 inhibition decreases cell cycle progression, migration and autophagy in bladder cancers. Int J Mol Sci. 2022;23:2154. 35216267 10.3390/ijms23042154 PMC8878685

[57] Zuo Q , Xu Q , Li Z , Luo D , Peng H , Duan Z . Kruppel‐like factor 5 enhances proliferation, lipid droplet formation and oxaliplatin resistance in colorectal cancer by promoting fatty acid binding protein 6 transcription. Anticancer Drugs. 2023; Publish Ahead of Print. 10.1097/CAD.0000000000001515 37067981

[58] Ma J , Xia LL , Yao XQ , et al. BARX2 expression is downregulated by CpG Island hypermethylation and is associated with suppressed cell proliferation and invasion of gastric cancer cells. Oncol Rep. 2020;43:1805‐1818. 32236603 10.3892/or.2020.7558 PMC7160541

[59] Chen H , Zhang M , Zhang W , et al. Downregulation of BarH‐like homeobox 2 promotes cell proliferation, migration and aerobic glycolysis through Wnt/beta‐catenin signaling, and predicts a poor prognosis in non‐small cell lung carcinoma. Thorac Cancer. 2018;9:390‐399. 29341468 10.1111/1759-7714.12593 PMC5832481

[60] Candler T , Kuhnen P , Prentice AM , Silver M . Epigenetic regulation of POMC; implications for nutritional programming, obesity and metabolic disease. Front Neuroendocrinol. 2019;54:100773. 31344387 10.1016/j.yfrne.2019.100773

[61] Yin A , Etcheverry A , He Y , et al. Integrative analysis of novel hypomethylation and gene expression signatures in glioblastomas. Oncotarget. 2017;8:89607‐89619. 29163774 10.18632/oncotarget.19171 PMC5685695

[62] Hahn A , Bode J , Alexander A , et al. Large‐scale characterization of the microvascular geometry in development and disease by tissue clearing and quantitative ultramicroscopy. J Cereb Blood Flow Metab. 2021;41:1536‐1546. 33043767 10.1177/0271678X20961854 PMC8217891

