# Supplementary figures and images for: Genome‐wide DNA methylation analysis identifies potent CpG signature for temzolomide response in non‐G‐CIMP glioblastomas with unmethylated MGMT promoter: MGMT ‐dependent roles of GPR81
Source: CNS Neurosci Ther. 2023 Oct 13;30(4):e14465. doi: 10.1111/cns.14465 (PMC11017469; doi:10.1111/cns.14465)

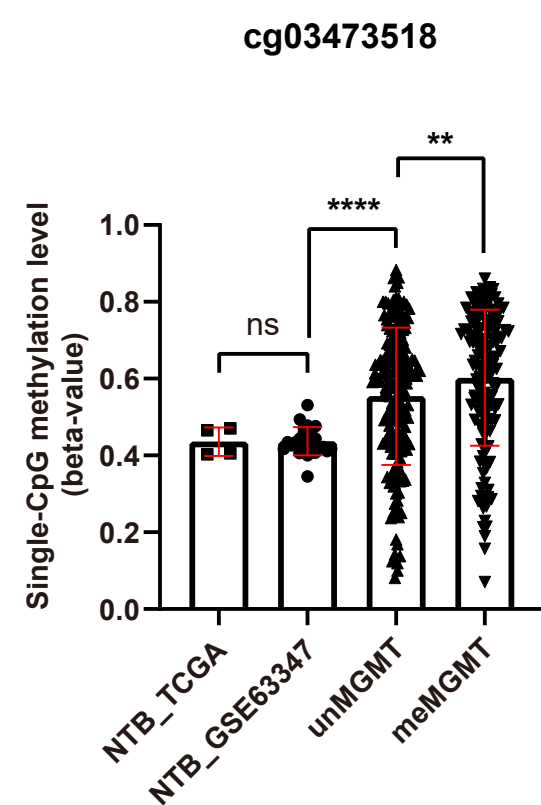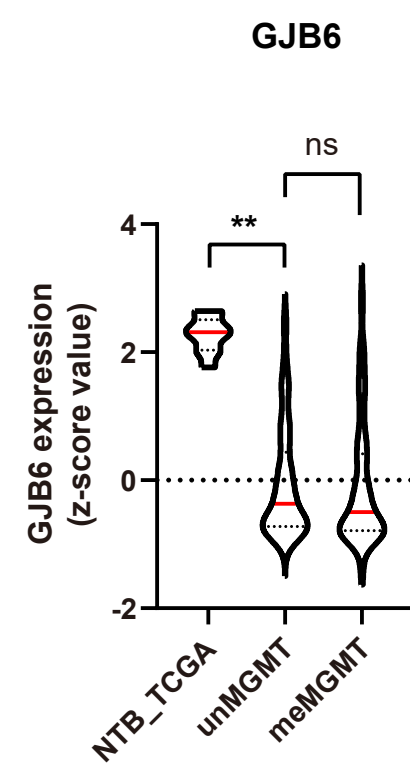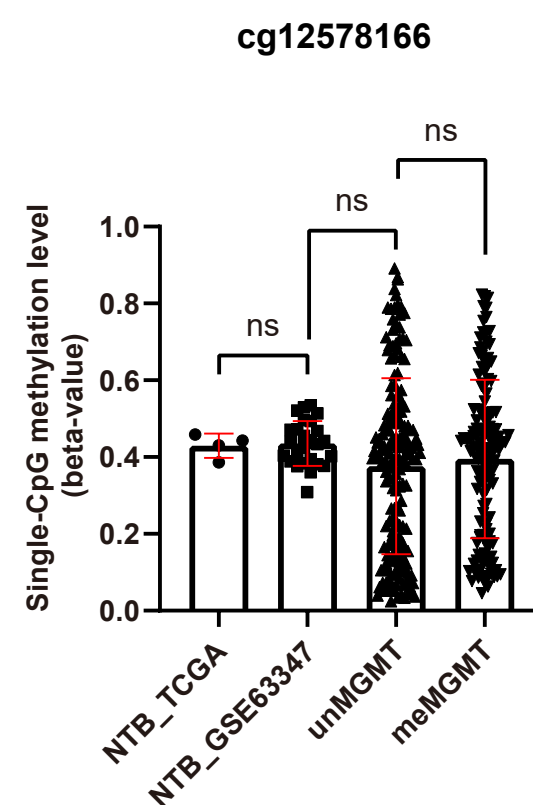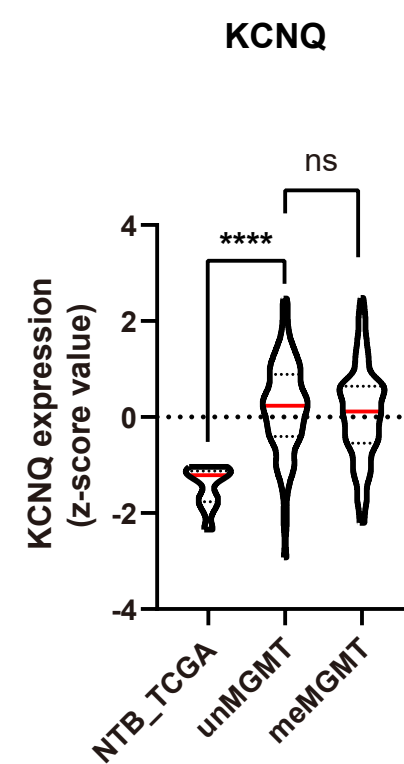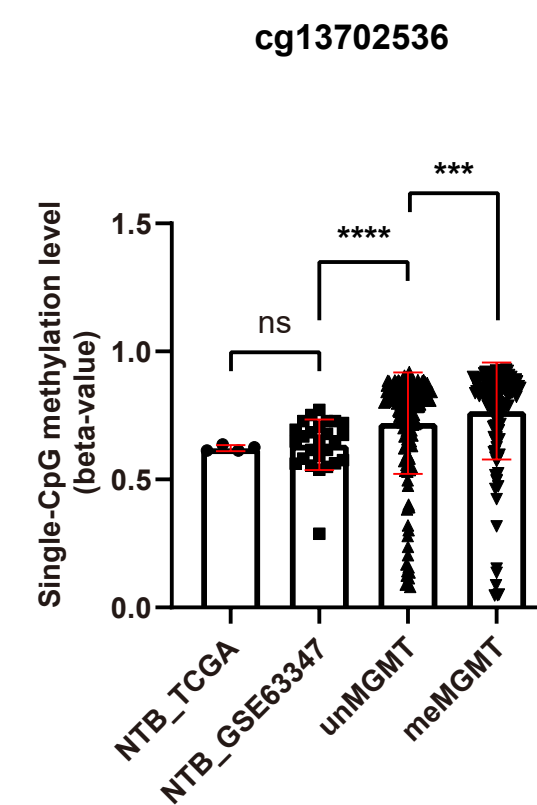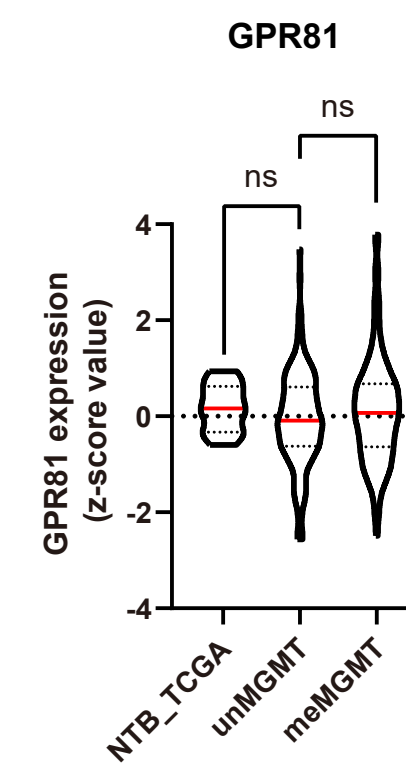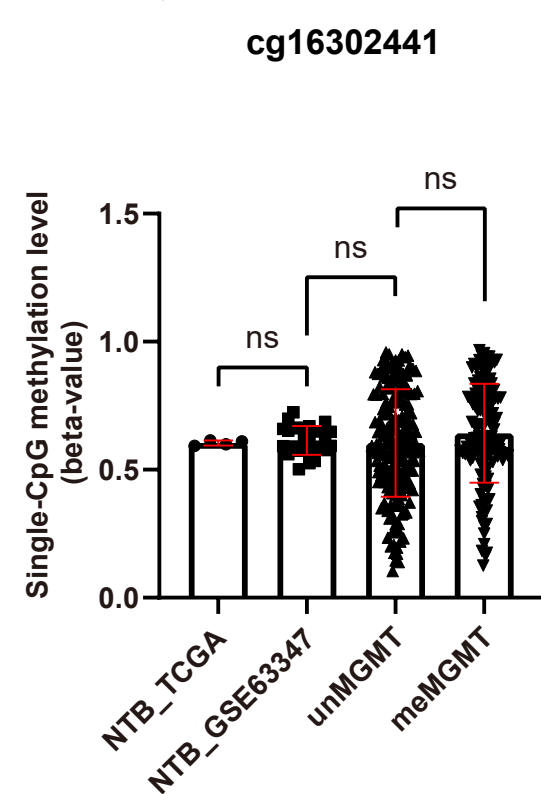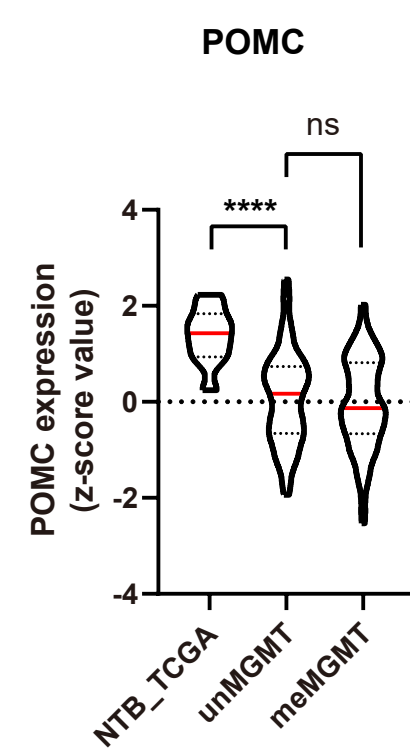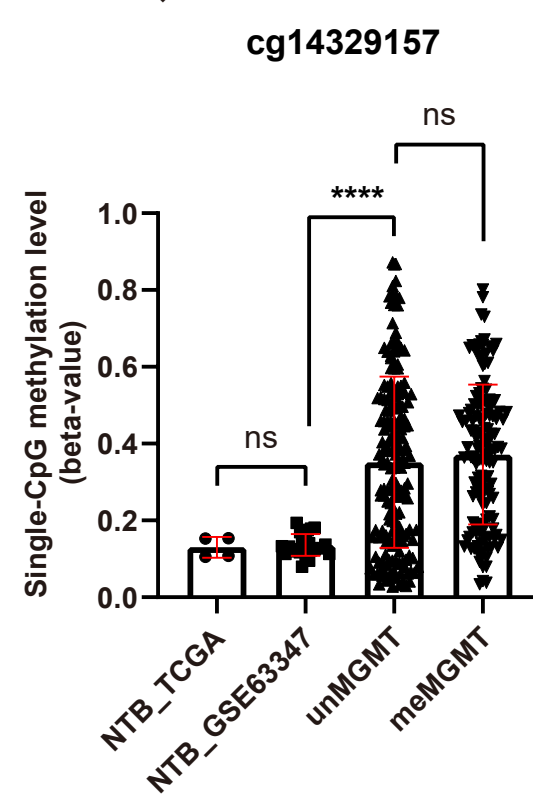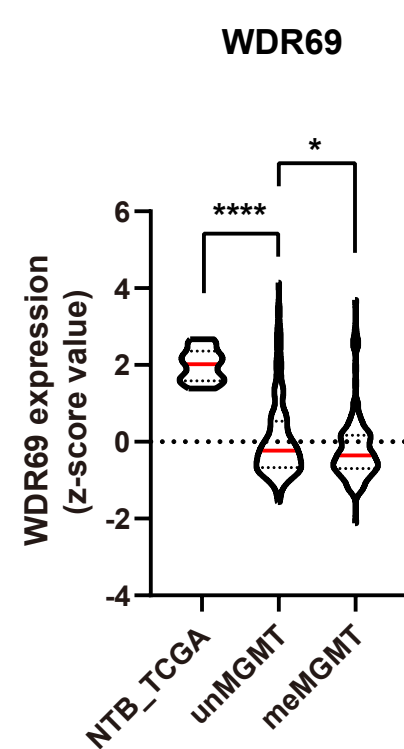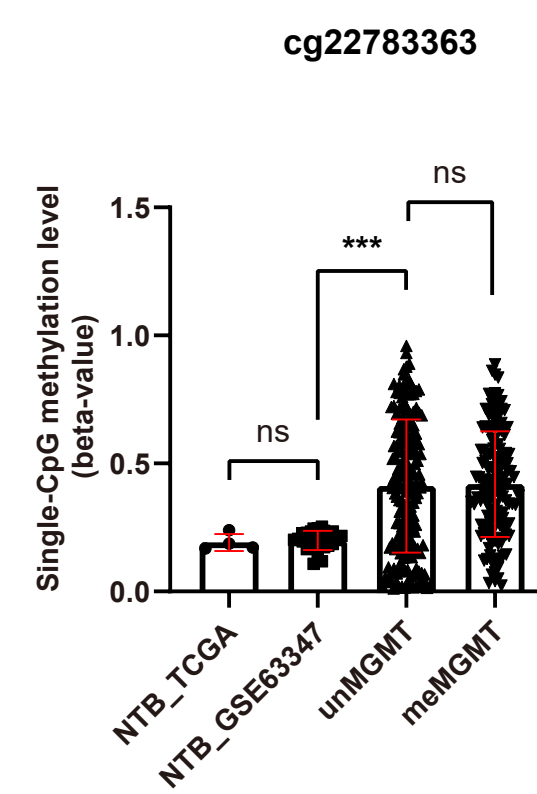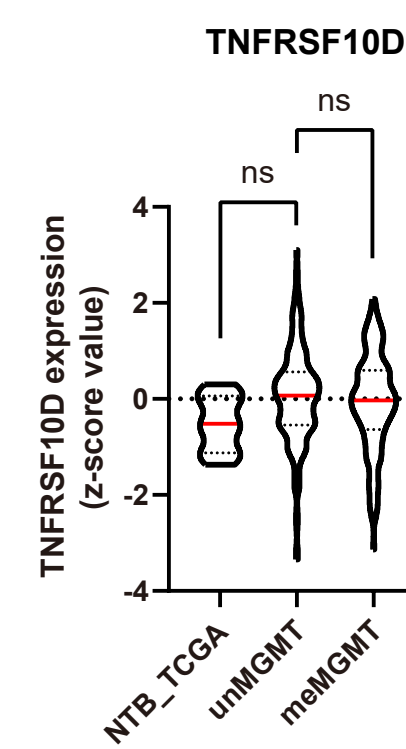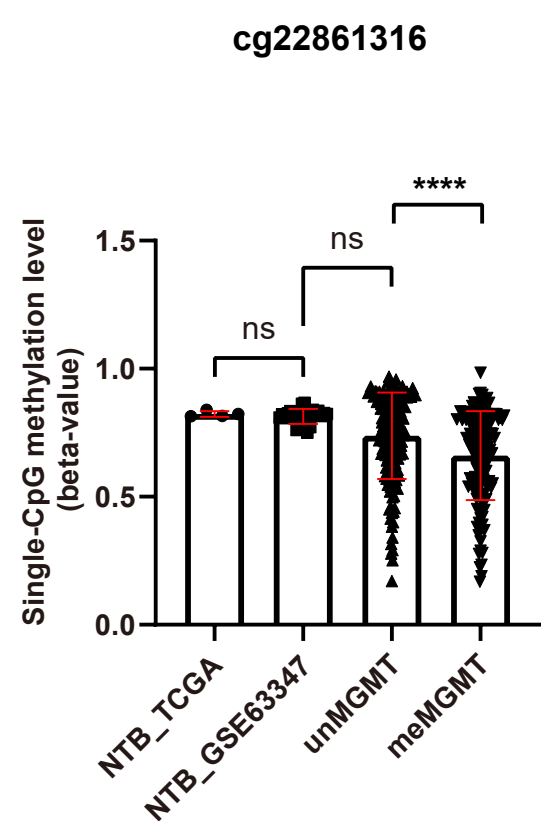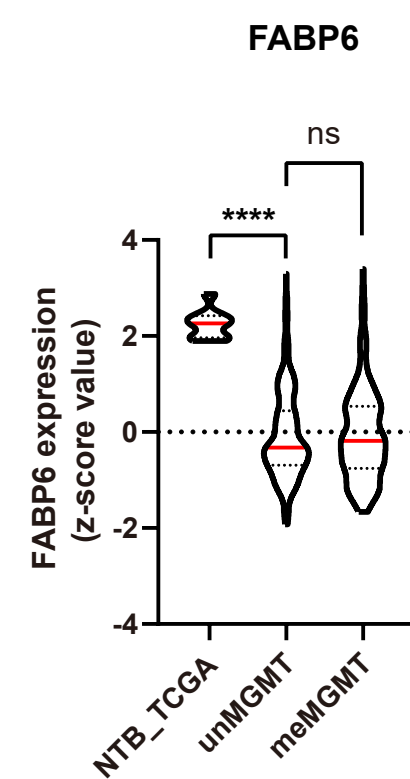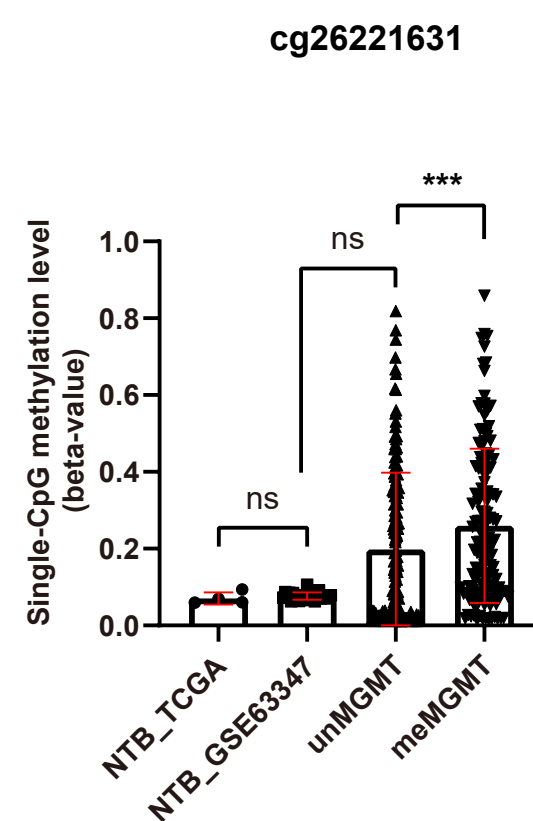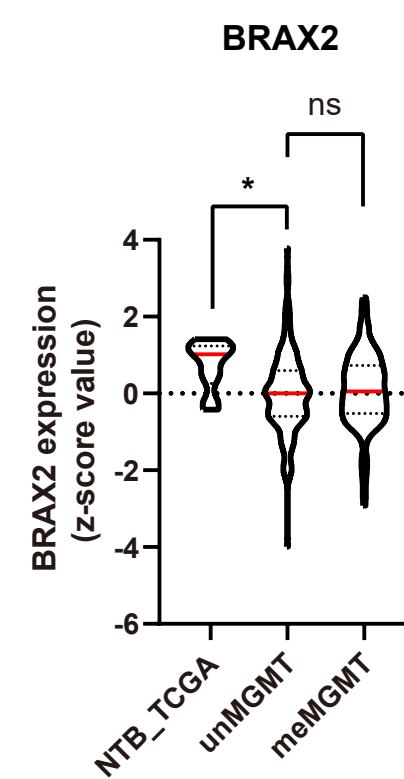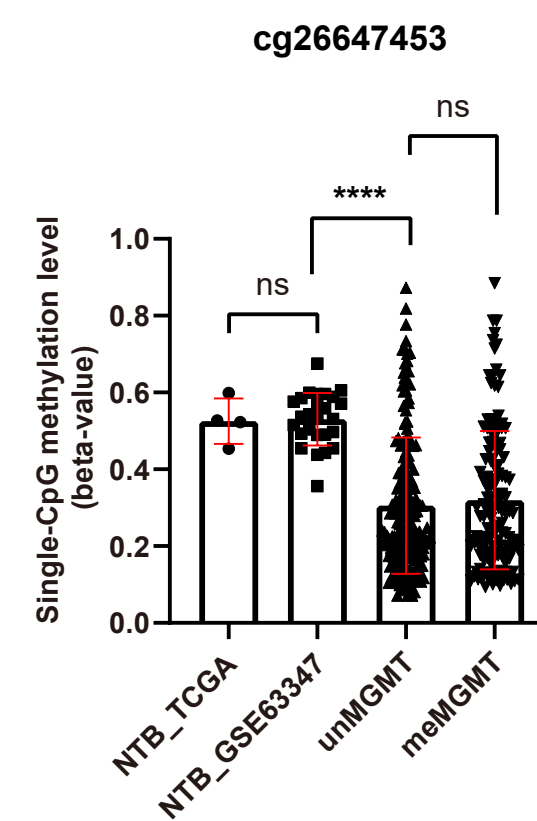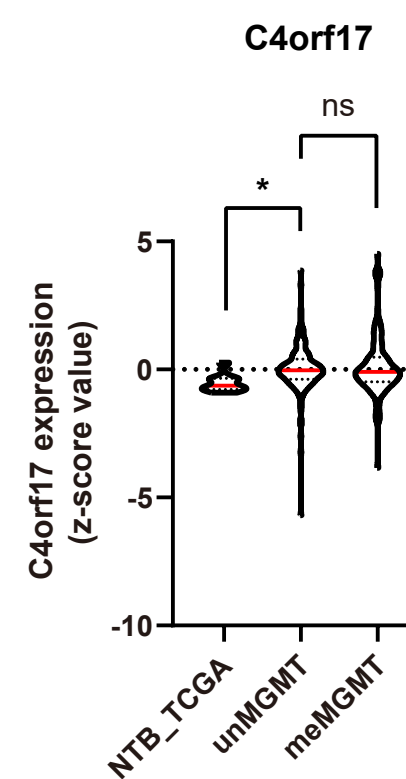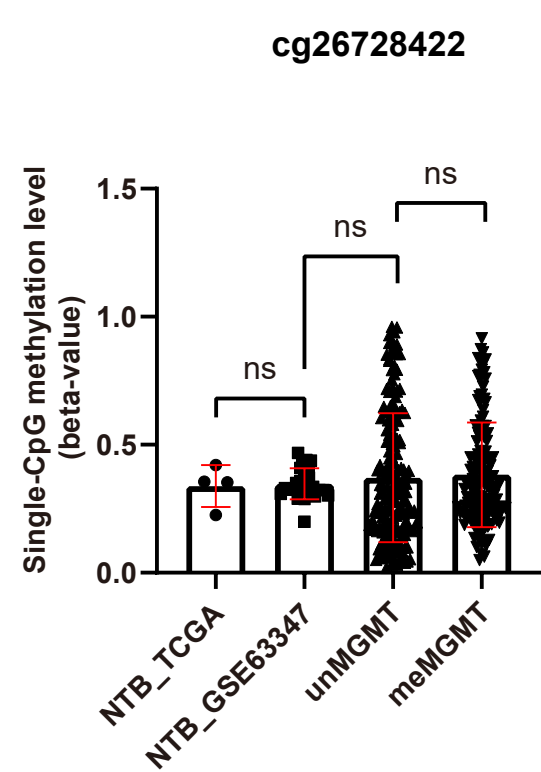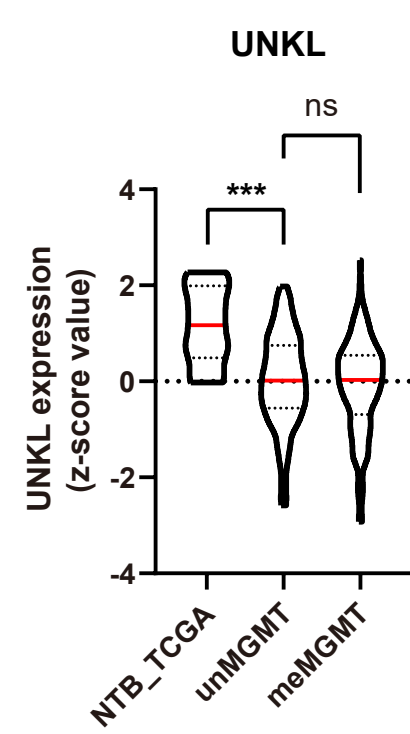

Supplement: Supplementary file 1 — Figure S1. [file CNS-30-e14465-s005.pdf]

# GSE68379 GBM cell lines

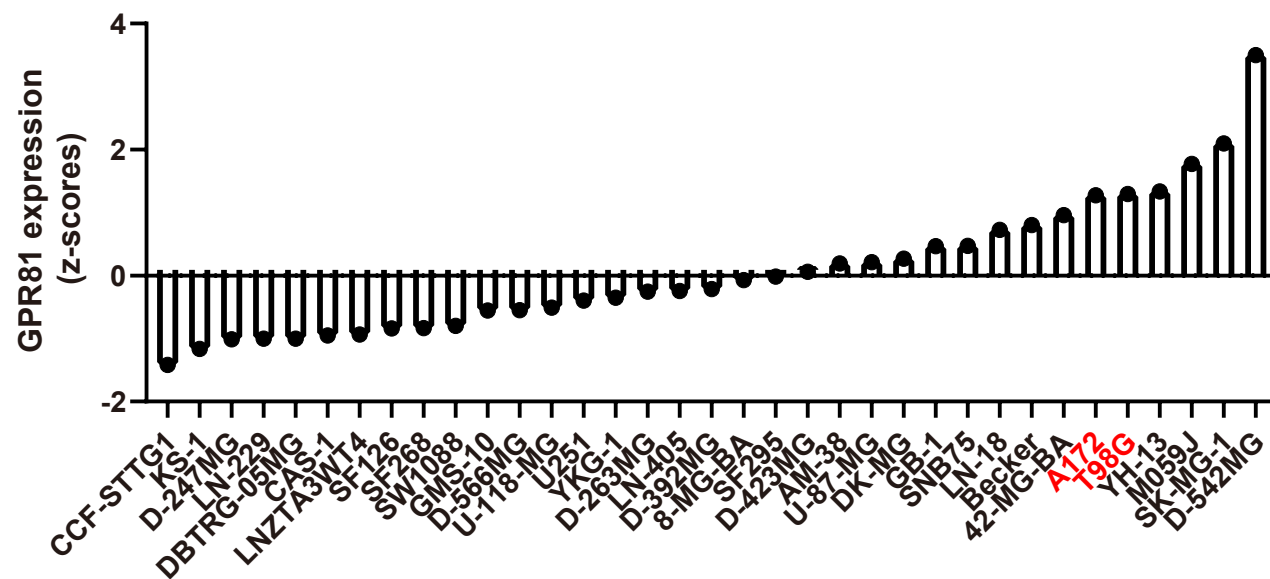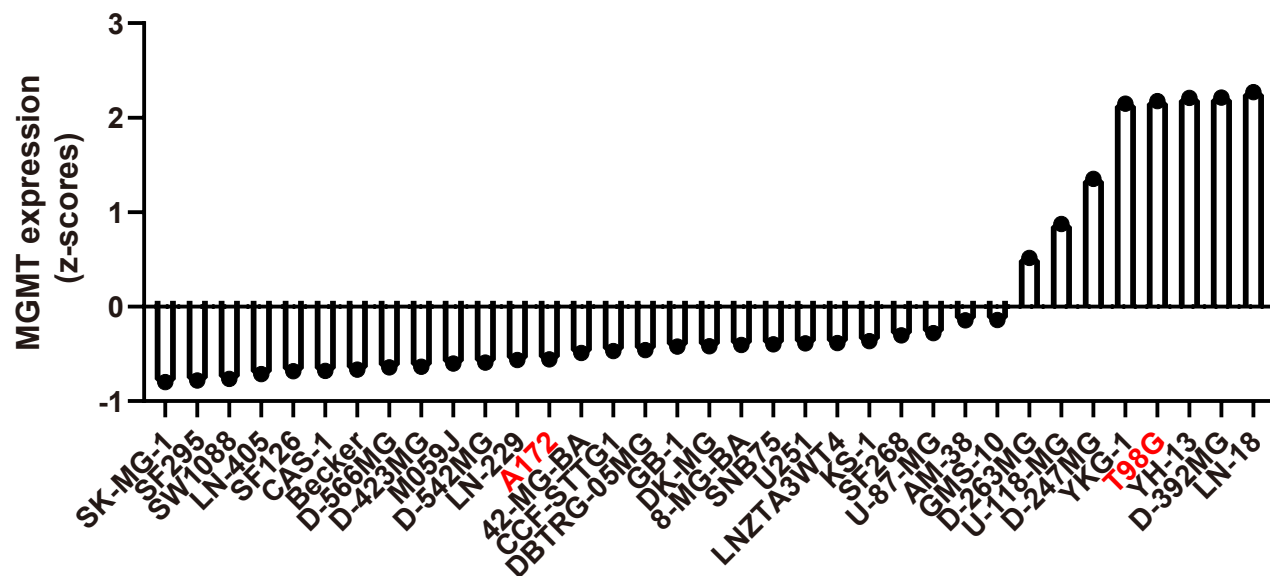

Supplement: Supplementary file 2 — Figure S2. [file CNS-30-e14465-s009.pdf]

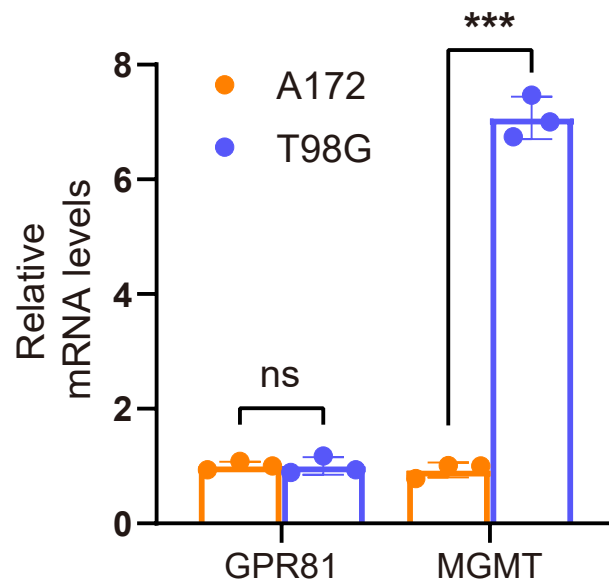

Supplement: Supplementary file 3 — Figure S3. [file CNS-30-e14465-s003.pdf]

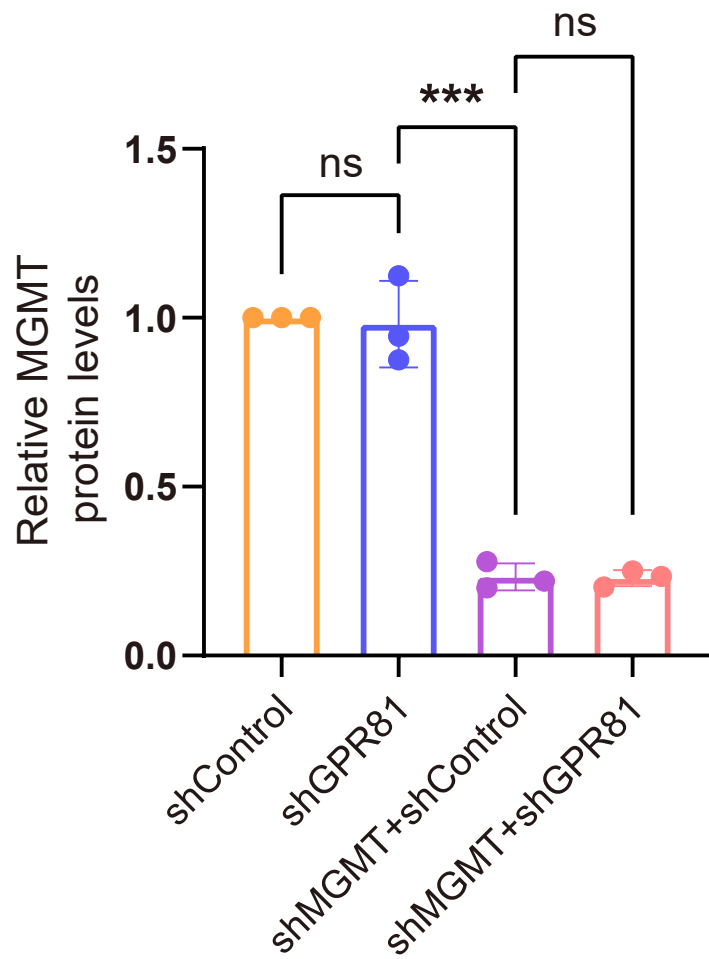

Supplement: Supplementary file 4 — Figure S4. [file CNS-30-e14465-s001.pdf]

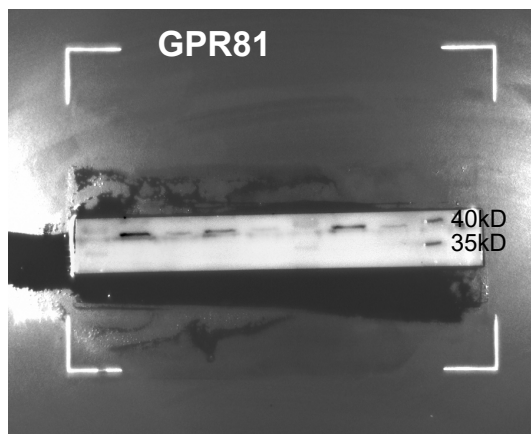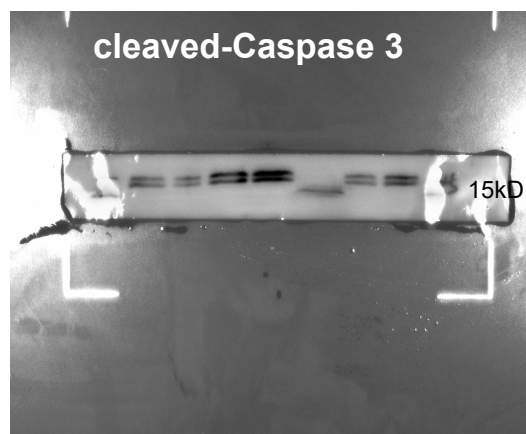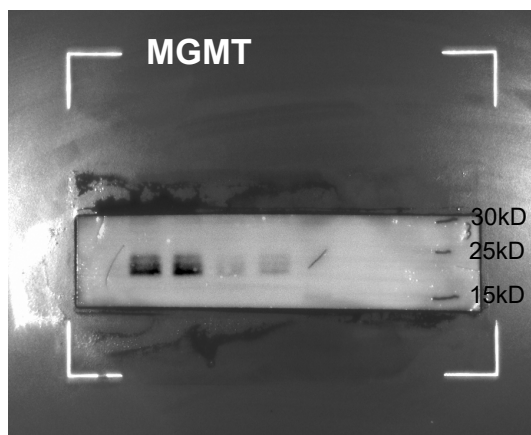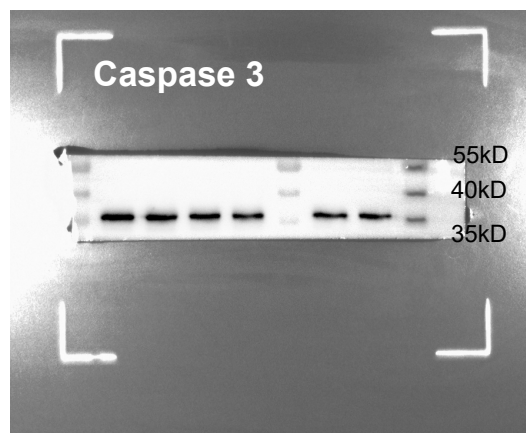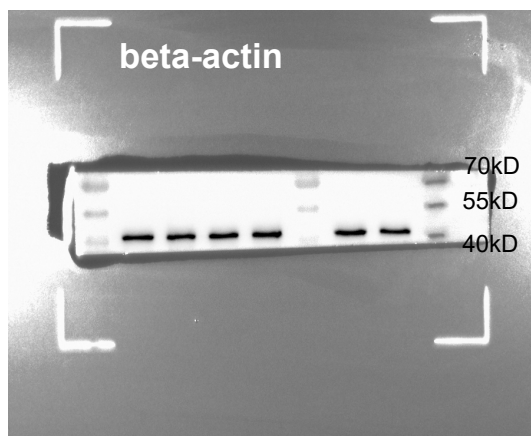

Supplement: Supplementary file 9 — Data S1. [file CNS-30-e14465-s002.zip › WB.pdf]

**GPR81**

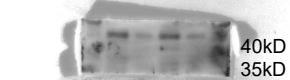

**cleaved-Caspase 3**

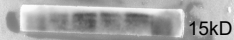

**MGMT**

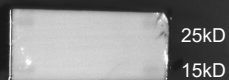

55kD  
40kD  
35kD

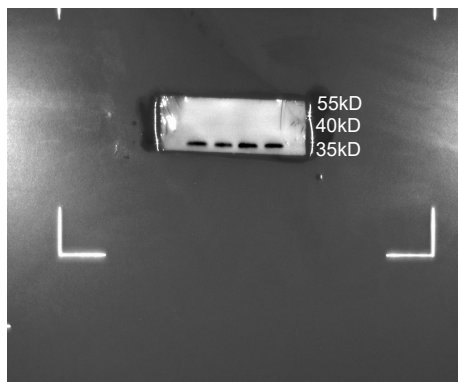

**beta-actin**

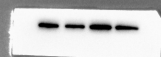

Supplement: Supplementary file 9 — Data S1. [file CNS-30-e14465-s002.zip › WB_R2.pdf]

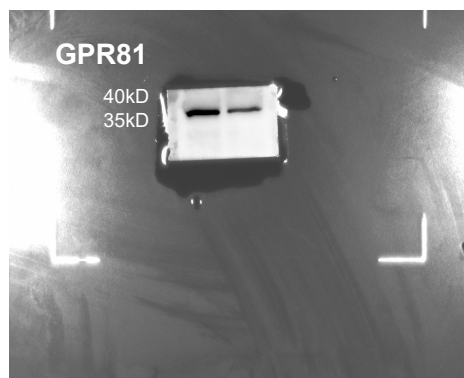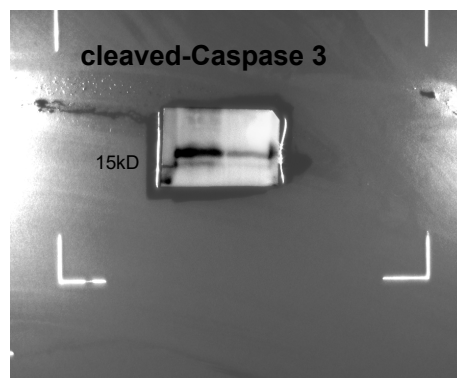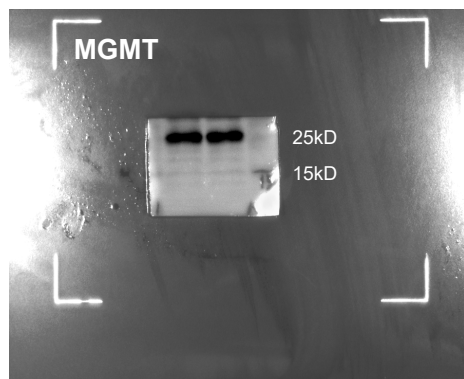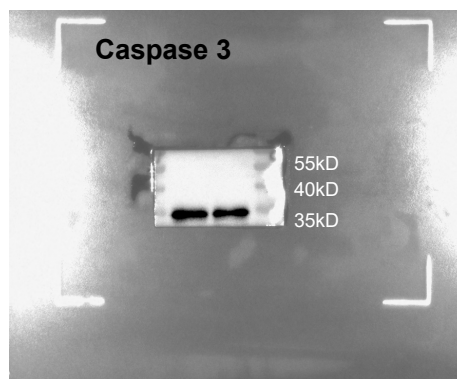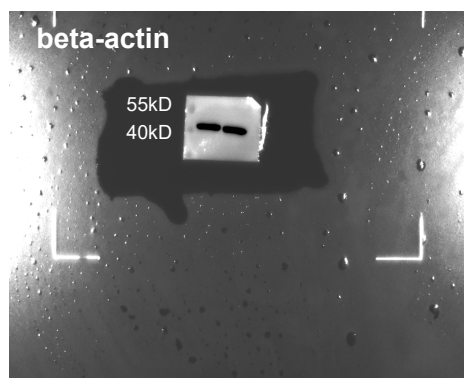

Supplement: Supplementary file 9 — Data S1. [file CNS-30-e14465-s002.zip › WB_R3.pdf]
