# Supplementary material for: Genome‐wide DNA methylation analysis identifies potent CpG signature for temzolomide response in non‐G‐CIMP glioblastomas with unmethylated MGMT promoter: MGMT ‐dependent roles of GPR81
Source: CNS Neurosci Ther. 2023 Oct 13;30(4):e14465. doi: 10.1111/cns.14465 (PMC11017469; doi:10.1111/cns.14465)
Supplement: Supplementary file 8 — Table S4. [file CNS-30-e14465-s004.docx]

Suppl. Table S4: Cox regression results for each indicated survival comparison

| Survival comparsion | Figures | HR | 95%CI | Pvalue |
| --- | --- | --- | --- | --- |
| *Discovery sets (TCGA+GEO, RT/TMZ, unMGMT)* | Figure 2B |  |  |  |
| Cluster 2 vs. 1 |  | 1.914 | 1.225-2.998 | **0.004** |
| Cluster 3 vs. 1 |  | 1.666 | 1.085-2.557 | **0.020** |
| Cluster 4 vs. 1 |  | 0.925 | 0.492-1.741 | 0.810 |
| G-CIMP+ vs. Cluster 1 |  | 0.226 | 0.031-1.656 | 0.143 |
| Cluster 3 vs. 2 |  | 0.811 | 0.542-1.214 | 0.308 |
| Cluster 4 vs. 2 |  | 0.427 | 0.213-0.856 | **0.017** |
| G-CIMP+ vs. Cluster 2 |  | 0.099 | 0.013-0.750 | **0.025** |
| Cluster 4 vs. 3 |  | 0.482 | 0.247-0.942 | **0.033** |
| G-CIMP+ vs. Cluster 4 |  | 0.108 | 0.015-0.805 | **0.030** |
| *Discovery sets (GSE60274, RT alone, unMGMT)* | Figure 2C |  |  |  |
| Cluster 2 vs. 1 |  | 0.817 | 0.165-4.033 | 0.804 |
| Cluster 3 vs. 1 |  | 2.850 | 0.516-15.732 | 0.229 |
| Cluster 4 vs. 1 |  | 0.872 | 0.175-4.347 | 0.868 |
| Cluster 3 vs. 2 |  | 2.562 | 0.225-29.121 | 0.448 |
| Cluster 4 vs. 2 |  | 2.562 | 0.225-29.121 | 0.448 |
| Cluster 4 vs. 3 |  | 0.618 | 0.055-6.995 | 0.697 |
| *Validation sets (RAUH, RT/TMZ, unMGMT)* | Figure 2E |  |  |  |
| Cluster 2 vs. 1 |  | 2.368 | 0.657-8.542 | 0.188 |
| Cluster 3 vs. 1 |  | 1.140 | 0.454-2.863 | 0.781 |
| Cluster 4 vs. 1 |  | 0.512 | 0.182-1.442 | 0.208 |
| Cluster 3 vs. 2 |  | 0.558 | 0.213-1.462 | 0.235 |
| Cluster 4 vs. 2 |  | 0.070 | 0.014-0.362 | **0.002** |
| Cluster 4 vs. 3 |  | 0.498 | 0.235-1.059 | 0.070 |
| *Validation sets (TCGA, RT alone, unMGMT)* | Figure 2F |  |  |  |
| Cluster 2 vs. 1 |  | 3.630 | 0.740-17.802 | 0.112 |
| Cluster 3 vs. 1 |  | 3.454 | 0.770-15.494 | 0.105 |
| Cluster 4 vs. 1 |  | 2.793 | 0.631-12.368 | 0.176 |
| G-CIMP+ vs. Cluster 1 |  | 0.342 | 0.053-2.189 | 0.257 |
| Cluster 3 vs. 2 |  | 0.746 | 0.288-1.932 | 0.546 |
| Cluster 4 vs. 2 |  | 0.994 | 0.371-2.663 | 0.990 |
| G-CIMP+ vs. Cluster 2 |  | 0.010 | 0.000-5.084 | 0.147 |
| Cluster 4 vs. 3 |  | 1.103 | 0.496-2.450 | 0.810 |
| G-CIMP+ vs. Cluster 4 |  | 0.015 | 0.000-1.420 | 0.071 |
| *TCGA (RT/TMZ)* | Figure 3A |  |  |  |
| high-risk vs. low-risk |  | 3.131 | 1.940-5.054 | **P<0.001** |
| meMGMT vs. low-risk |  | 0.702 | 0.468-1.054 | 0.088 |
| G-CIMP+ vs. low-risk |  | 0.379 | 0.175-0.821 | **0.014** |
| meMGMT vs. high-risk |  | 0.259 | 0.165-0.407 | **P<0.001** |
| G-CIMP+ vs. high-risk |  | 0.116 | 0.041-0.331 | **P<0.001** |
| G-CIMP+ vs. meMGMT |  | 0.596 | 0.284-1.253 | 0.172 |
| *GEO (RT/TMZ)* | Figure 3A |  |  |  |
| high-risk vs. low-risk |  | 4.965 | 2.714-9.083 | **P<0.001** |
| meMGMT vs. low-risk |  | 0.639 | 0.394-1.036 | 0.069 |
| G-CIMP+ vs. low-risk |  | 0.291 | 0.109-0.775 | **0.014** |
| meMGMT vs. high-risk |  | 0.151 | 0.085-0.268 | **P<0.001** |
| G-CIMP+ vs. high-risk |  | 0.056 | 0.013-0.248 | **P<0.001** |
| G-CIMP+ vs. meMGMT |  | 0.541 | 0.231-1.264 | 0.156 |
| *GSE60274 (RT alone)* | Figure 3B |  |  |  |
| high-risk vs. low-risk |  | 0.936 | 0.304-2.883 | 0.908 |
| meMGMT vs. low-risk |  | 0.751 | 0.315-1.786 | 0.517 |
| G-CIMP+ vs. low-risk |  | 1.473 | 0.171-12.711 | 0.724 |
| meMGMT vs. high-risk |  | 0.632 | 0.221-1.893 | 0.412 |
| G-CIMP+ vs. high-risk |  | 1.072 | 0.109-10.574 | 0.953 |
| G-CIMP+ vs. meMGMT |  | 1.848 | 0.215-15.897 | 0.576 |
| *GSE22891 (RT/TMZ)* | Figure 3C |  |  |  |
| high-risk vs. low-risk |  | 3.127 | 1.146-8.537 | **0.026** |
| meMGMT vs. low-risk |  | 0.585 | 0.251-1.360 | 0.213 |
| G-CIMP+ vs. low-risk |  | 0.026 | 0.000-17.006 | 0.269 |
| meMGMT vs. high-risk |  | 0.203 | 0.084-0.493 | **P<0.001** |
| G-CIMP+ vs. high-risk |  | 0.022 | 0.000-4.560 | 0.161 |
| G-CIMP+ vs. meMGMT |  | 0.042 | 0.000-32.236 | 0.349 |
| *GSE50923 (RT/TMZ)* | Figure 3C |  |  |  |
| high-risk vs. low-risk |  | 6.891 | 2.409-19.710 | **P<0.001** |
| meMGMT vs. low-risk |  | 1.020 | 0.460-2.262 | 0.962 |
| G-CIMP+ vs. low-risk |  | 0.515 | 0.135-1.964 | 0.331 |
| meMGMT vs. high-risk |  | 0.170 | 0.070-0.412 | **P<0.001** |
| G-CIMP+ vs. high-risk |  | 0.082 | 0.010-0.665 | **0.019** |
| G-CIMP+ vs. meMGMT |  | 0.530 | 0.157-1.790 | 0.306 |
| GSE60274 (RT/TMZ) | Figure 3C |  |  |  |
| high-risk vs. low-risk |  | 4.716 | 1.388-16.022 | **0.013** |
| meMGMT vs. low-risk |  | 0.260 | 0.092-0.733 | **0.011** |
| G-CIMP+ vs. low-risk |  | 0.144 | 0.018-1.171 | 0.070 |
| meMGMT vs. high-risk |  | 0.067 | 0.014-0.329 | **0.001** |
| G-CIMP+ vs. high-risk |  | 0.015 | 0.000-4.145 | **0.015** |
| G-CIMP+ vs. meMGMT |  | 0.962 | 0.261-3.539 | 0.953 |
| RAUH (RT/TMZ) | Figure 3D |  |  |  |
| high-risk vs. low-risk |  | 2.203 | 1.163-4.173 | **0.015** |
| meMGMT vs. low-risk |  | 0.612 | 0.325-1.153 | 0.128 |
| G-CIMP+ vs. low-risk |  | 0.309 | 0.041-2.326 | 0.254 |
| meMGMT vs. high-risk |  | 0.313 | 0.153-0.638 | **0.001** |
| G-CIMP+ vs. high-risk |  | 0.029 | 0.000-3.772 | 0.154 |
| G-CIMP+ vs. meMGMT |  | 0.602 | 0.079-4.577 | 0.624 |
| TCGA (RT alone) | Figure 3E |  |  |  |
| high-risk vs. low-risk |  | 0.922 | 0.481-1.768 | 0.806 |
| meMGMT vs. low-risk |  | 0.832 | 0.448-1.545 | 0.560 |
| G-CIMP+ vs. low-risk |  | 0.052 | 0.007-0.408 | **0.005** |
| meMGMT vs. high-risk |  | 0.922 | 0.495-1.719 | 0.799 |
| G-CIMP+ vs. high-risk |  | 0.116 | 0.026-0.523 | 0.005 |
| G-CIMP+ vs. meMGMT |  | 0.132 | 0.030-0.570 | 0.007 |
| *All cohorts (RT/TMZ)* | Figure 3F |  |  |  |
| high-risk vs. low-risk |  | 3.356 | 2.447-4.603 | **P<0.001** |
| meMGMT vs. low-risk |  | 0.663 | 0.504-0.873 | **0.003** |
| G-CIMP+ vs. low-risk |  | 0.347 | 0.198-0.606 | **P<0.001** |
| meMGMT vs. high-risk |  | 0.230 | 0.169-0.313 | **P<0.001** |
| G-CIMP+ vs. high-risk |  | 0.087 | 0.040-0.190 | **P<0.001** |
| G-CIMP+ vs. meMGMT |  | 0.587 | 0.344-1.003 | **0.051** |
| *All cohorts (RT alone)* | Figure 3G |  |  |  |
| high-risk vs. low-risk |  | 0.951 | 0.546-1.655 | 0.859 |
| meMGMT vs. low-risk |  | 0.776 | 0.461-1.278 | 0.319 |
| G-CIMP+ vs. low-risk |  | 0.146 | 0.043-0.503 | **0.002** |
| meMGMT vs. high-risk |  | 0.772 | 0.452-1.319 | 0.344 |
| G-CIMP+ vs. high-risk |  | 0.157 | 0.045-0.546 | **0.004** |
| G-CIMP+ vs. meMGMT |  | 0.219 | 0.067-0.718 | **0.012** |
| *TCGA (RT/TMZ)* | Figure 4A |  |  |  |
| high-risk vs. low-risk |  | 1.420 | 0.972-2.075 | 0.070 |
| meMGMT vs. low-risk |  | 0.698 | 0.484-1.008 | 0.055 |
| G-CIMP+ vs. low-risk |  | 0.414 | 0.202-0.847 | **0.016** |
| meMGMT vs. high-risk |  | 0.551 | 0.381-0.797 | **0.002** |
| G-CIMP+ vs. high-risk |  | 0.357 | 0.175-0.729 | **0.005** |
| G-CIMP+ vs. meMGMT |  | 0.602 | 0.311-1.166 | 0.133 |
| *RAUH (RT/TMZ)* | Figure 4B |  |  |  |
| high-risk vs. low-risk |  | 2.361 | 1.256-4.437 | **0.008** |
| meMGMT vs. low-risk |  | 0.828 | 0.447-1.532 | 0.547 |
| G-CIMP+ vs. low-risk |  | 0.239 | 0.031-1.838 | 0.169 |
| meMGMT vs. high-risk |  | 0.408 | 0.205-0.811 | **0.011** |
| G-CIMP+ vs. high-risk |  | 0.025 | 0.000-3.116 | 0.134 |
| G-CIMP+ vs. meMGMT |  | 0.477 | 0.063-3.610 | 0.473 |
| *TCGA (RT alone)* | Figure 4C |  |  |  |
| high-risk vs. low-risk |  | 0.813 | 0.428-1.543 | 0.526 |
| meMGMT vs. low-risk |  | 0.681 | 0.370-1.254 | 0.217 |
| G-CIMP+ vs. low-risk |  | 0.044 | 0.000-194.354 | 0.465 |
| meMGMT vs. high-risk |  | 0.928 | 0.503-1.714 | 0.812 |
| G-CIMP+ vs. high-risk |  | 0.044 | 0.000-418.264 | 0.503 |
| G-CIMP+ vs. meMGMT |  | 0.045 | 0.000-520.973 | 0.516 |
